# Long read genome assemblies complemented by single cell RNA-sequencing reveal genetic and cellular mechanisms underlying the adaptive evolution of yak

**DOI:** 10.1038/s41467-022-32164-9

**Published:** 2022-09-06

**Authors:** Xue Gao, Sheng Wang, Yan-Fen Wang, Shuang Li, Shi-Xin Wu, Rong-Ge Yan, Yi-Wen Zhang, Rui-Dong Wan, Zhen He, Ren-De Song, Xin-Quan Zhao, Dong-Dong Wu, Qi-En Yang

**Affiliations:** 1 Key Laboratory of Adaptation and Evolution of Plateau Biota, Northwest Institute of Plateau Biology, Chinese Academy of Sciences, Xining, Qinghai 810001 China; 2 University of Chinese Academy of Sciences, Beijing, 100049 China; 3 State Key Laboratory of Genetic Resources and Evolution, Kunming Institute of Zoology, Chinese Academy of Sciences, Kunming, Yunnan China; 4 Center for Excellence in Animal Evolution and Genetics, Chinese Academy of Sciences, Kunming, Yunnan 650223 China; 5 Kunming Natural History Museum of Zoology, Kunming Institute of Zoology, Chinese Academy of Sciences, Kunming, Yunnan 650223 China; 6 Center for Animal Disease Control and Prevention, Yushu, Qinghai 815000 China; 7 Qinghai Key Laboratory of Animal Ecological Genomics, Northwest Institute of Plateau Biology, Chinese Academy of Sciences, Xining, 810001 China

**Keywords:** Functional genomics, Genomics, Animal physiology

## Abstract

Wild yak (*Bos mutus*) and domestic yak (*Bos grunniens*) are adapted to high altitude environment and have ecological, economic, and cultural significances on the Qinghai-Tibetan Plateau (QTP). Currently, the genetic and cellular bases underlying adaptations of yak to extreme conditions remains elusive. In the present study, we assembled two chromosome-level genomes, one each for wild yak and domestic yak, and screened structural variants (SVs) through the long-read data of yak and taurine cattle. The results revealed that 6733 genes contained high-FST SVs. 127 genes carrying special type of SVs were differentially expressed in lungs of the taurine cattle and yak. We then constructed the first single-cell gene expression atlas of yak and taurine cattle lung tissues and identified a yak-specific endothelial cell subtype. By integrating SVs and single-cell transcriptome data, we revealed that the endothelial cells expressed the highest proportion of marker genes carrying high-FST SVs in taurine cattle lungs. Furthermore, we identified pathways which were related to the medial thickness and formation of elastic fibers in yak lungs. These findings provide new insights into the high-altitude adaptation of yak and have important implications for understanding the physiological and pathological responses of large mammals and humans to hypoxia.

## Abstract (web summary)

The genetic bases of yak adaptations to extreme conditions remains elusive. This study compares yak and cattle at a genomic and transcriptomic level, revealing a new type of endothelial cell and candidate genes related with elastic fiber formation in yak lungs that might contribute to high altitude adaptation.

## Introduction

Yak is one of the largest ruminants living in the areas with the highest average altitude in the world and physiologically adapted to hypoxic and cold environment. The domestic yak normally lives between 3000 and 5000 m above sea level while the wild yak, the ancestor of domestic yak, inhabits at elevations from 4000 to 6000 m on the QTP^1–5^. Archaeological and molecular evidences revealed that wild yak was domesticated at least 7300 years ago^6–9^. Although significant morphologic differences exist between wild and domestic yak^1^, both species share genetic features of high-altitude adaptation and are considered excellent models for studying hypoxia tolerance in large mammals.

In 2012, the genome of a female domestic yak was sequenced using the Illumina-based whole-genome shotgun method^6^. In 2020, the genome of a female wild yak was assembled with the Illumina data^7^, and chromosome-scale genomes of female domestic yak were constructed using long-read sequencing and chromatin interaction technologies^10,11^. However, previous studies on yak have primarily focused on single nucleotide variants to reveal the genomic diversity and historical population dynamics^6,12–18^. Recent studies showed that structural variants (SVs), such as insertions, deletions, duplications, inversions, and translocations, are widely present in genomes^19^ and provide an extensive source of genetic variations for identifying candidate genes involved in the regulation of critical biological processes^20,21^. The availability of high-quality reference genomes for taurine cattle, which were obtained using long-read sequencing technologies, has enabled the dissection of genetic basis of complex traits^22^. Assembling high-quality and complete reference genomes of wild and domestic yak is fundamental for deciphering the molecular mechanisms underpinning adaptation of yak and related species to extreme high-altitude environment on the QTP. Unfortunately, due to quality-related issues of the wild yak reference genome, SVs and SV-related genes in wild yak and domestic yak have yet to be mapped and compared with those in taurine cattle genome in detail.

Unique genomic features and precisely controlled gene expression ensure the physiological adaptation of animals to high altitude. Non-native mammals are prone to altitude sickness, mainly manifested as pulmonary hypertension and right ventricular hypertrophy after their exposure to acute or long-term hypoxia^23–25^. Yak acquired specific anatomical and physiological characteristics to survive the oxygen-poor air and the harsh environment. It is known that their blood, lung, and heart systems have been evolved to meet the challenge at high altitude^23^. Wild and domestic yak are adapted to hypoxia by increasing hemoglobin content, red blood cell count and hematocrit^26^. The lung and heart weights of yak are higher than those of age-matched taurine cattle^25^. Among these tissues, the lung is the interface between environment and body, therefore it plays a pivotal role in high-altitude adaptation. Yak developed intense pulmonary blood vessels, which increase the oxygen exchange rate of pulmonary artery blood vessels and help relieve pulmonary artery pressure^23^. By comparing transcriptomic profiles among different tissues across species, it was reported that the lung exhibited adaptive transcriptional changes and expressed a higher number of positively selected genes^27^. Despite these findings, the cellular components, and gene expression dynamics of lung tissues in animals that are adapted to high altitude remain to be explored at single cell level.

In the present study, we used Nanopore sequencing and Hi-C data to assemble the high-quality genomes of a wild yak and a domestic yak. We used an alignment-based strategy to compare long-read sequencing data with taurine cattle, to identify SVs associated with high altitude adaptation. We also constructed the single-cell atlas of yak and taurine cattle lungs using single-cell transcriptome sequencing (scRNA-seq). Integrated genomics and transcriptomics analyses revealed a new subtype of endothelial cells and uncovered a list of genes and pathways that were associated with the development of unique structures in yak lung. All together, these data provided important information to understand genetic and cellular mechanisms underlying the adaptive evolution of yak.

## Results

### Chromosome level genomes of wild and domestic yak

DNAs from an adult male wild yak and an adult male domestic yak (Supplementary Fig. 1) were extracted for sequencing and genome assembly. A total of 157.17 Gb data from wild yak and 191.17 Gb data from domestic yak were generated using Nanopore long-reading sequencing technology and wtdbg2 program^28^ (Supplementary Tables 1–3). The contig N50 of wild yak genome was 39.41 Mbp with 2283 contigs, while it reached 46.79 Mbp with 1451 contigs for domestic yak genome (Table 1; Supplementary Tables 4–6). In order to further improve the accuracy of genome assembly, Illumina whole genome shotgun (WGS) short reading data (wild yak: 114.38-fold and domestic yak: 57.11-fold genome coverage) were utilized to correct remaining errors. The contigs were then anchored to chromosome model using Hi-C data (wild yak: 109.26-fold and domestic yak: 143.56-fold genome coverage) (Supplementary Tables 2 and 3). Finally, the genome of wild yak was successfully assembled with the size of 2.63 Gbp and 2058 contigs in 30 chromosomes. The genome of domestic yak reached 2.61 Gbp, with 1284 contigs in 30 chromosomes. Both genomes were close to the estimated size of wild yak (2.8 Gb) and domestic yak (3.1 Gb) (Table 1).

**Table 1:** Genome assembly statistics

	Domestic yak	Wild yak
Sequencing technology	Nanopore; Illumina HiSeq; Hi-C	Nanopore; Illumina HiSeq; Hi-C
Number of scaffolds	1314	2275
Scaffold N50/Mb	104.02	103.90
Number of contigs	1451	2283
Contig N50/Mb	44.91	38.28
Total size/Gb	2.61	2.63
Number of genes	23,143	22,931

Next, we evaluated the sequence consistency and integrity of the two newly assembled genomes. The comparison rate of all small fragments was 99.35% and the coverage rate was 99.19% in wild yak while those for domestic yak were 99.18% and 99.59%, respectively (Supplementary Table 7), indicating high quality in their sequence consistency and integrity. Because the heterozygous SNP ratios were 0.1371% and 0.1590%, and the homozygous SNP ratio were 0.0009% and 0.0004% for the wild yak and domestic yak genomes, respectively (Supplementary Table 8), we concluded that our genome assemblies had a relatively high single-base accuracy. CEGMA assessment showed that 94.76% CEGs (Core Eukaryotic Genes) were assembled in both wild and domestic yak genomes (Supplementary Table 9). BUSCO evaluation indicated that 93.3% and 93.2% of the complete single-copy genes in wild and domestic yak, respectively, were assembled from 4104 orthologous single-copy genes (Supplementary Table 10). Merqury evaluation results indicated that the qv value of domestic yak was 30.1682, while that of wild yak was 30.5424. All these results indicated relatively completed assemblies of both wild and domestic yak genomes.

### Annotation for repetitive sequences and genes in wild and domestic yak

The two genomes of wild yak and domestic yak were then annotated for repetitive sequences and genes. The predicted de novo repeat sequence library was integrated with the homologous repeat sequence database (Repbase), and then the genomes were annotated for repeats with RepeatMasker^29^. The genome of wild yak carried 45.81% repeat sequences while the genome of domestic yak contained 45.67% repeat sequences (Supplementary Tables 11–14; Supplementary Figs. 2 and 3). Six related ruminant species, including buffalo (*Bubalus_bubalis*), cattle (*Bos_taurus*), sheep (*Ovis_aries*), goat (*Capra_hircus*), European bison (*Bison_bonasus*), and reindeer (*Rangifer_tarandus*), were selected for gene homology prediction. A total of 22,931 and 23,143 genes were predicted in the wild and domestic yak genomes, respectively (Supplementary Figs. 4–7; Supplementary Tables 15–18). By comparing the protein sequences which were predicted based on gene structures to library, 95.4% and 95.3% of the genes were estimated to be functional in wild and domestic yak, respectively (Supplementary. Figs. 8 and 9; Supplementary Table 19). Using the known library as a reference, the ncRNAs in both wild and domestic yak genomes were also screened (Supplementary Tables 20 and 21).

### Identification of structural variants by comparing the yak and taurine cattle genomes

**Fig. 1:**
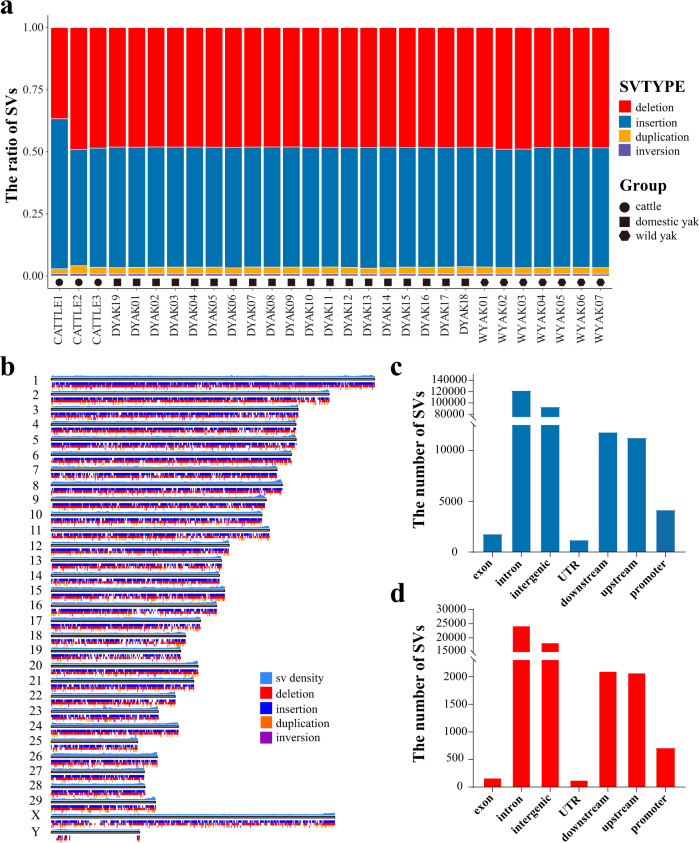
Detection and characterization of structural variants (SVs) in yak and cattle genomes. **a** Stacked bar graph showing the number and type of SV from the 29 individuals. **b** The chromosomal landscape of SVs. **c** The histogram showing the number of SVs counted from all SVs located in exon, intron, intergenic, UTR, downsteam, upsteam, and promoter. **d** The histogram showing the number of SVs counted from the high-FST SVs located in exon, intron, intergenic, UTR, downsteam, upsteam, and promoter. Source data are provided as a Source Data file.

The long-read sequencing has proven to be powerful for the identification of SVs^30–32^. Here, we applied NGMLR-cuteSV pipeline to detect the SVs in 3 taurine cattle, 19 domestic yak, and 7 wild yak based on the comparison with the taurine cattle genome^33,34^ (Fig. 1a; Supplementary Fig. 10). A total of 74,279 deletions, 79,467 insertions, 3,464 duplications, and 867 inversions were detected (Fig. 1b; Supplementary Fig. 11). Because the length of the translocation is 0 in the cuteSV result, we did not include the translocation for further analysis. We annotated all SVs by their positions on ARS-UCD1.2 and found 122,103 SVs (50.58%) were in intronic regions, and 4,163, 93,243, 1,785, 1,197 and 23,076 SVs were in promoter, intergenic, exonic, UTR or the 150 bp upstream and downstream flanking regions of genes, respectively (Fig. 1c). In order to further determine the SVs that likely participate in high altitude adaptation, we calculated the F-statistics (FST) for all SVs between taurine cattle and yak, and selected SVs with the top 0.5% FST values (High-FST SVs) as candidate sites. Interestingly, we found that FST values of these candidate SVs were 1 (FST = 1) and this accounts for 12.5% of the SVs (Supplementary Fig. 12). Among these high-FST SVs, 24,468 SVs are (10.14% of all SVs) in the intronic and 712, 18,483, 174, 136 and 4,187 SVs were in promoter, intergenic, exonic, UTR or the 150 bp upstream and downstream flanking regions of genes, respectively (Fig. 1d). Of these, 11 genes contained deletions causing frameshifts in the ORF (Supplementary Table 22). We then annotated the 6733 genes carrying high-FST SVs and 897 genes carrying special SVs located in the exonic and promoter regions (Supplementary Data 1).

### Integrating transcriptome and chromatin accessibility data to reveal potentially functional SVs

**Fig. 2:**
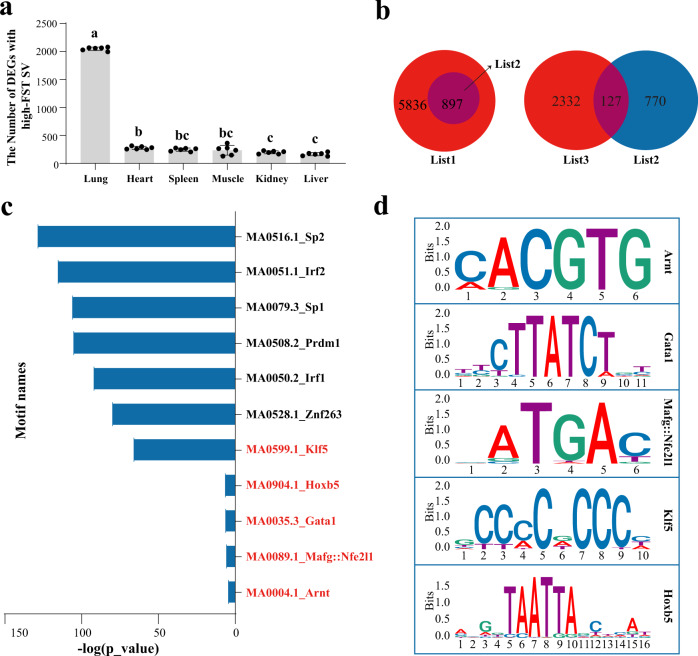
Screening of candidate genes in high-altitude adaptation of yak. **a** The number of DEGs in lung, heart, spleen, kidney, muscle, and liver between taurine cattle (*n* = 3 biologically independent samples) and yak (*n* = 4 biologically independent samples) with high-FST SVs. 5 cattle and 5 yak lung tissue transcriptome data were used for analysis. Different letters (a, b, c) denotes statistical significance at *P* < 0.05 (One-way ANOVA with Tukey’s test for multiple comparisons; *F* = 1722; *P* < 0.0001). The error bars respectively are 2049.17 ± 28.76, 278.17 ± 24.79, 247.17 ± 25.96, 236.83 ± 79.19, 198.83 ± 23.64 and 166.33 ± 27.20 in six tissues. **b** The venn diagrams of three gene lists. List1: Genelist annotated with high-FST SVs. List2: Genelist annotated with high-FST SVs located in exon and promoter. List3: Differentially expressed genes between domestic yak and taurine cattle in lungs. **c** The motif enrichment results of peak located in the promoter with high-FST SVs. The *p* values were calculated using Fisher’s exact test. **d** The sequence information of five special motifs. Source data are provided as a Source Data file.

Many SVs lie in the noncoding sequences, including promoter and UTR regions, and they are associated with the expression of nearby genes. To interrogate the potential roles of SVs in influencing gene expression, we examined differentially expressed genes (DEGs) between six domestic yak and taurine cattle organs including heart, lung, kidney, spleen, muscle, and liver, using RNA-sequencing data from 48 samples (Supplementary Table 23). It should be noted that we did not find SVs in the yak-specific gene (Supplementary Table 24), therefore, only the homologous genes of yak and cattle were used to calculate DEGs. We found 2049.17 ± 28.76, 278.17 ± 24.79, 247.17 ± 25.96, 236.83 ± 79.19, 198.83 ± 23.64 and 166.33 ± 27.20 DEGs carrying high FST SVs in the heart, lung, kidney, spleen, muscle, and liver, respectively. Results of one-way ANOVA and Tukey’s multiple comparisons showed that the number in the lung was different from those in other tissues examined (*P* < *0.0001*, *F* = 1722) (Fig. 2a; Supplementary Data 2). We next conducted the Chi-square test for detecting relationships between the DEGs and SV-carrying genes in six tissues, and the results suggested that the identified SVs were associated with the differential expression of genes in all tissues analyzed (All *p* values were <0.01; χ^2^ value was from 9.15 in the heart to 41.14 in the lung) (Fig. 2a; Supplementary Data 3). By integrating relevant genomic variation data, we discovered that 127 genes carrying high-FST SVs within the exonic and promoter regions were differentially expressed in lungs of the taurine cattle and domestic yak (Fig. 2b; Supplementary Table 25). In addition, we performed motif enrichment analysis on the peak of the promoter regions carrying high-FST SVs using the MEME suite. As a result, 632 peaks were identified in the promoter regions carrying high-FST SVs and 446 motifs were obtained after enrichment analysis (Fig. 2c; Supplementary Data 4). Particularly, 5 transcription factors that were previously identified to be important for the hypoxia response were enriched (Fig. 2d). ARNT participates in the hypoxia-inducible factor (HIF) signaling pathway^35^. GATA1 directs the expression of genes involved in heme biosynthesis, erythropoietin signaling, and anti-apoptotic and proliferation pathways, and is also required for EPOR expression^36^. MAFG interacts with HIF-1α and controls hypoxia response^37^. KLF5 is crucial for hypoxia-induced vascular remodeling and its expression is directly regulated by HIF-1α^38^. HOXB5 controls airway and alveolar development through cell-cell communication between the mesenchyme and epithelial cell compartments^39^. Together, these data revealed that the SVs in the promoters of hypoxia-related genes have potential functions in gene expression regulation.

### Single cell RNA-seq analysis of lung tissues of domestic yak and taurine cattle

**Fig. 3:**
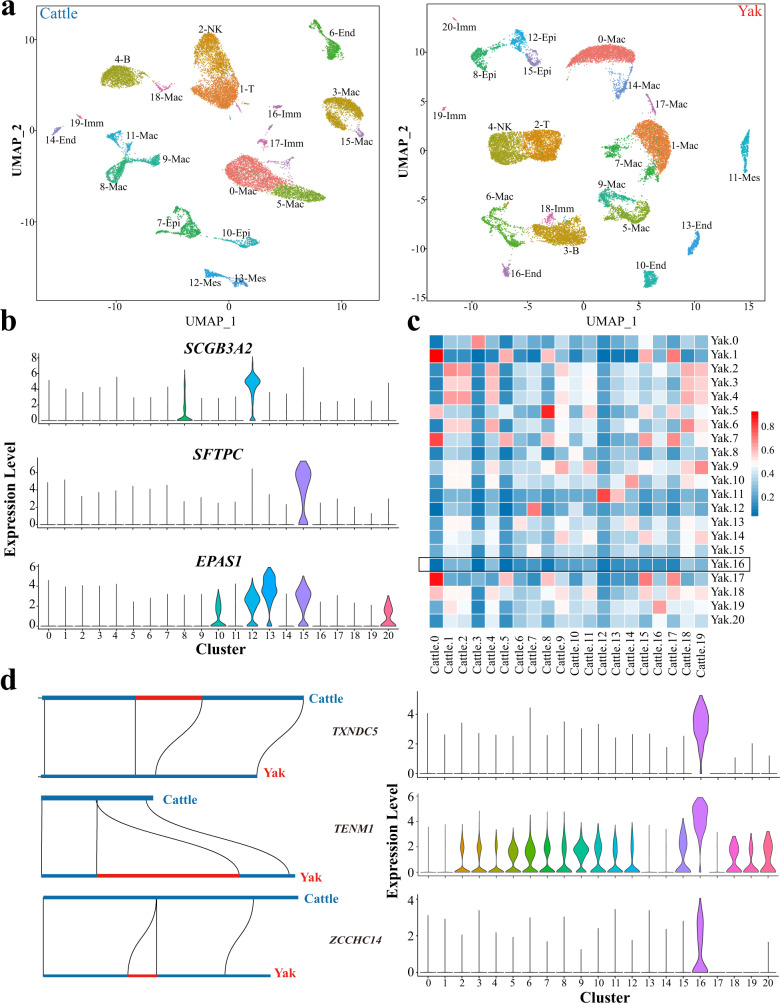
Single cell RNA sequencing (ScRNA-seq) identified different cell clusters in taurine cattle and domestic yak lungs. **a** UMAP clustering of various cell clusters in taurine cattle (left, *n* = 5 biologically independent samples) and domestic yak lung (right, *n* = 5 biologically independent samples). **b** Violin diagram of the expressions of special marker genes in the domestic yak lung (*n* = 5 biologically independent samples). **c** Heatmap of the pearson correlation coefficients between different clusters of domestic yak (*n* = 5 biologically independent samples) and taurine cattle (*n* = 5 biologically independent samples). The boxes show specific cell clusters being weakly correlated. **d** Schematic diagram about SV and violin plots of *TXNDC5*, *TENM1* and *ZCCHC14* in the domestic yak lung (*n* = 5 biologically independent samples). Source data are provided as a Source Data file.

Although the lung is one of the most important organs mediating physiological adaptations to hypoxia, the diversity of cell populations and gene expression profiles at the single cell level has not been studied in lungs of high-altitude animals. To that end, we digested the domestic yak (*n* = 5) and taurine cattle lung tissues (*n* = 5) and obtained single cells for RNA-seq analysis (Supplementary Fig. 13). After two library constructions, a total of 31579 domestic yak cells and 27951 taurine cattle cells were harvested and sequenced. After cell filtration and integration of two samples, we used Uniform Manifold Approximation and Projection (UMAP) dimensionality reduction strategy^40^, a coarse clustering of the transcriptomic data at the resolution of 0.5 to subdivide the single-cell transcriptomic data into 21 and 20 cell clusters in domestic yak (yak 0–20) and taurine cattle (cattle 0–19) respectively (Fig. 3a; Supplementary Figs. 14 and 15). Four main cell types were identified in the domestic yak lung, including epithelial cells (7.5%, expressing *SEC14L3*, *SCGB1A1*, and *SFTPC*), endothelial cells (8.0%, expressing *MMRN1*, *S100A1*, and *CCL21*), mesenchymal cells (2.9%, expressing *COL1A1*, *COL3A1*, and *DCN*), and immune cells (81.6%, expressing *CD3E*, *IL1RN*, and *CCL5*) (Fig. 3a; Supplementary Fig. 14). Similarly, these four cell types of epithelial cells (8.6%, expressing *SEC14L3*, *SCGB3A2*, and *SFTPC*), endothelial cells (5.3%, expressing *MMRN1*, *CALCRL*, and *CCL21*), mesenchymal cells (4.5%, expressing *COL1A1*, *COL3A1*, and *DCN*), and immune cells (81.6%, expressing *CD3E*, *CCL5*, and *IL1RN*) were also present in the taurine cattle lung (Fig. 3a; Supplementary Fig. 15). These four cell types were present in mouse lung, suggesting that the major cell components of lung tissues were conserved among species^41^. *SFTPC* and *SCGB3A2*, the two genes that were specifically expressed in lung tissues, exhibited their enrichments in yak15 and yak12 cell clusters (epithelial cells) (Fig. 3b). *EPAS1* gene, which is related to high altitude adaptation^42^, was enriched in yak13 clusters (endothelial cells) (Fig. 3b). Furthermore, we uncovered new markers for all cell types, for instance, *CYP2B6* and *TPPP3* in epithelial cells, *EPAS1* in endothelial cells, *COL3A1* in mesenchymal cells, and *MS4A1*, and *BRB* in immune cells (Supplementary Figs. 14 and 15).

Based on the results of the Pearson correlation coefficients^43,44^, we concluded that the relatively correlations were weak between the yak16, a type of endothelial cell, and all the 20 clusters of taurine cattle cells (the r-value of each cell subgroup of the cattle <0.31) (Fig. 3c; Supplementary Data 5). The Yak16 cluster likely corresponded to a new population of cells that highly expressed *TXNDC5*, *TENM1*, and *ZCCHC14* which contained high-FST intronic SVs (Fig. 3d; Supplementary Data 1). *TXNDC5* (Thioredoxin Domain Containing 5) promotes fibroblast activation, proliferation, and excessive ECM (extracellular matrix) production by enhancing TGF-β signaling activity through post-translational stabilization and up-regulation of Tgfbr1 in lung fibroblasts^45^ (Fig. 3d). *TENM1* (Teneurin Transmembrane Protein 1), which is involved in neural development^46^ (Fig. 3d). *ZCCHC14* (zinc finger, CCHC domain containing) deletion in mice causes microcephaly, intellectual disability, bilateral vesicoureteral reflux, and bulbar venous malformations^47^ (Fig. 3d; Supplementary Fig. 16).

### Identification of gene expression patterns and cellular components in domestic yak and taurine cattle lungs

**Fig. 4:**
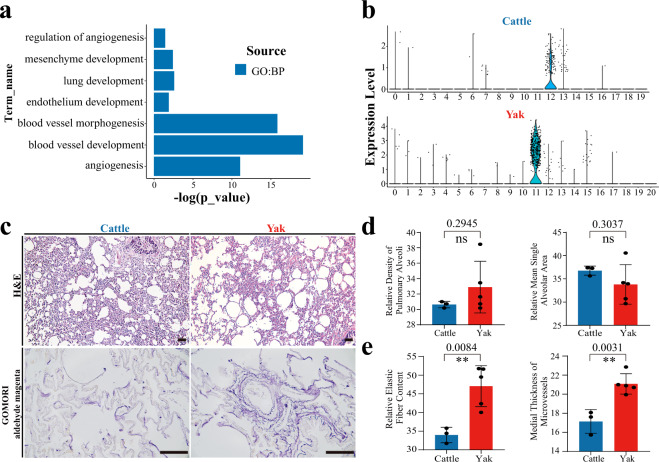
Gene expression and histological analysis of the domestic yak and taurine cattle lungs. **a** Enrichment analysis of DEGs in different mesenchymal cell cluster, statistical analysis was performed using a hypergeometric test. **b** Violin plots of the patterns of *LOX* expressions in the domestic yak (*n* = 5 biologically independent samples) and taurine cattle (*n* = 5 biologically independent samples) lung at the single cell level. **c** H&E and elastic fiber staining in the domestic yak (*n* = 5 biologically independent samples) and taurine cattle lung (*n* = 3 biologically independent samples). Scale bar = 50 μm. **d** Quantifications of the density of pulmonary alveoli and relative mean single alveolar area in the domestic yak (*n* = 5 biologically independent samples, 33.81 ± 3.80 in relative mean single alveolar area, 32.89 ± 3.00 in relative density of pulmonary alveoli) (ns, no significance; T-test, two-tailed) and taurine cattle lung (*n* = 3 biologically independent samples, 36.76 ± 0.80 in relative mean single alveolar area, 30.64 ± 0.34 in relative density of pulmonary alveoli) (ns, no significance; T-test; two-tailed). **e** Measurements of medial thickness of microvessels and elastic fiber content in the domestic yak (*n* = 5 biologically independent samples, 21.08 ± 0.96 in medial thickness of microvessels, 47.06 ± 4.91 in relative elastic fiber content) (ns, no significance; T-test, two-tailed) and taurine cattle lung (*n* = 3 biologically independent samples, 17.14 ± 1.02 in medial thickness of microvessels, 33.99 ± 1.69 in relative elastic fiber content) (ns, no significance; T-test, two-tailed). (T-test; **p < 0.01; T-test, two-tailed). Source data are provided as a Source Data file.

In addition to the differences in cell components, we proposed that differential gene expressions in the same cell population may also contribute to the adaptation of yak. We found that mesenchymal cells (yak-11 and cattle-12&13 clusters) had 1024 DEGs (Supplementary Data 6). Gene enrichment analysis of these DEGs identified pathways of blood vessel development and morphogenesis (Fig. 4a; Supplementary Data 7). Blood vessel development and angiogenesis in lung are crucial for the establishment of adaptive mechanisms in hypoxic environment^23^. The *LOX* gene, which is localized at the elastin/microfibril interface in aorta and cross-links both elastin and collagen, and involved in elastic fiber assembly^48^, exhibited enrichment in domestic yak mesenchymal cells (Fig. 4b). Indeed, histological examination of lung tissues using hematoxylin and eosins (H&E) staining uncovered that the density of pulmonary alveoli and relative mean single alveolar area did not differ, however, the medial thickness of micro-vessels was significantly thicker in domestic yak than taurine cattle, which was likely related to difference in blood vessel development and mesenchymal cell proliferation between two species (T-test; *P* = 0.0031; two-tailed; *t* = 4.767; df = 8) (Fig. 4c–e). Importantly, quantitative analysis using Gomori staining revealed that elastic fiber content was significantly higher in domestic yak than taurine cattle (T-test; *P* = 0.0084; two-tailed; *t* = 3.860; df = 8) (Fig. 4c–e).

**Fig. 5:**
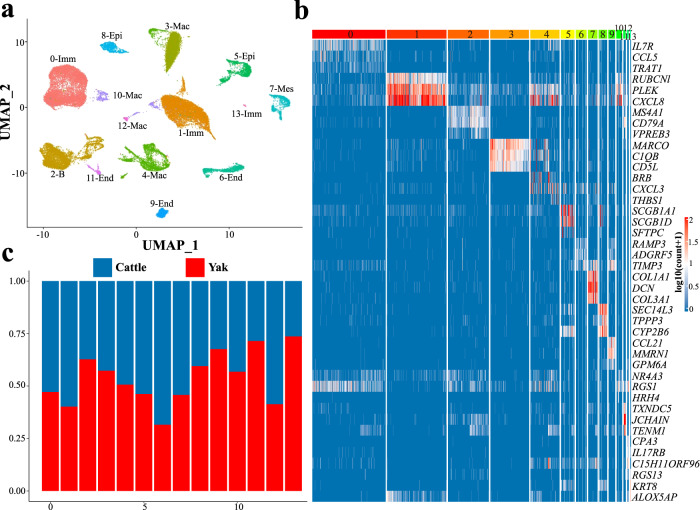
Integrative analysis of single-cell RNA-seq data in the domestic yak and taurine cattle lungs. **a** UMAP clustering of different cell clusters. **b** Heatmap of the expressions of specific marker genes in each cluster. **c** Histogram of the ratios of different types of cells within each cell cluster in the domestic yak (*n* = 5 biologically independent samples) and taurine cattle lungs (*n* = 5 biologically independent samples). Source data are provided as a Source Data file.

To examine the differences in cellular components and gene expression features, we used the canonical correlation analysis (CCA) to perform an integrated analysis of single-cell RNA-seq data from domestic yak and taurine cattle^49,50^. A further UMAP analysis identified six cell clusters at the resolution of 0.1 (Fig. 5a). Four major cell types were identified, including epithelial cells (*n* = 1639, expressing *SEC14L3* and *CYP2B6*), endothelial cells (*n* = 1095, expressing *MMRN1* and *CCL21*), immune cells (*n* = 16,255, expressing *PLEK* and *IL7R*), and mesenchymal cells (*n* = 737, expressing *DCN* and *COL1A1*) (Fig. 5b). Out of them, cluster 11 constituted 71.44 % of all cells in domestic yak while cluster 6 contained 68.46% of all cells in taurine cattle, both were endothelial cells (Fig. 5c).

### Integrating genomic variants and single-cell RNA-sequencing data to reveal genetic underpinning of adaptation in domestic yak

**Fig. 6:**
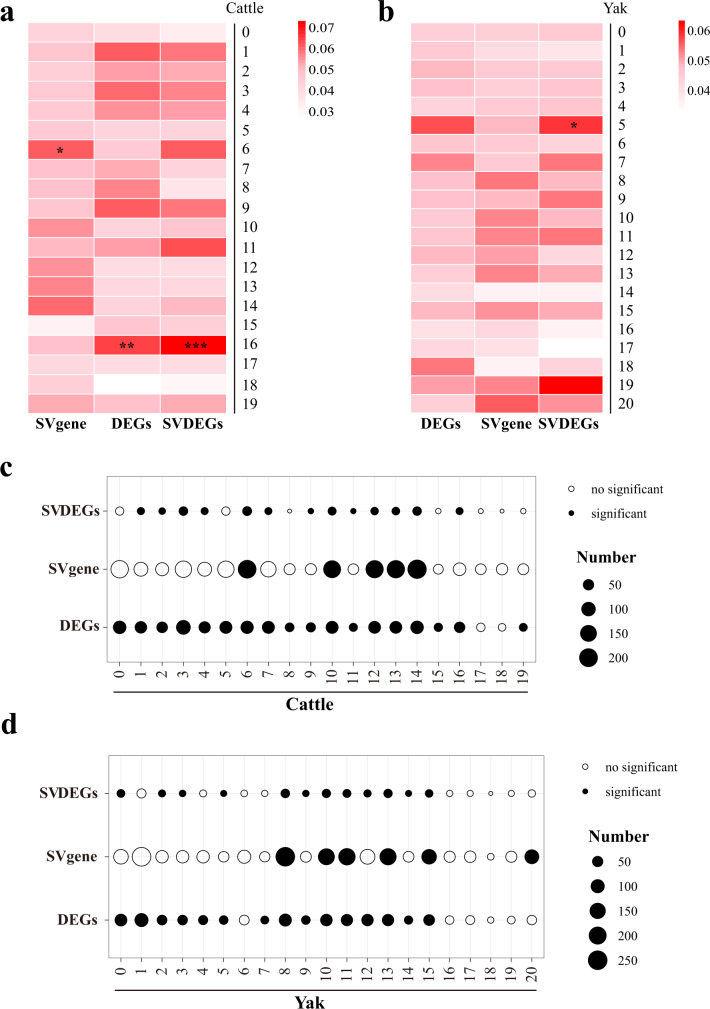
Enrichment of SVs in different cell clusters identified by scRNA-seq. **a** The heatmap shows the normalized ratio of SVgene, DEGs and SVDEGs in the marker genes of each cluster in the taurine cattle lung (*n* = 5 biologically independent samples) (Note: *P = *0.0243*; **P = 0.0038*;* ***P = 0.0007; T-test; two-tailed). **b** The heatmap shows the normalized ratio of SVgene, DEGs, and SVDEGs in the marker genes of each cluster in the domestic yak lung (*n* = 5 biologically independent samples) (Note: *P = 0.0337; T-test; two-tailed). **c** The bubble chart shows the overlap significance of the three gene lists and the marker genes of each cluster in the taurine cattle (*n* = 5 biologically independent samples) lung respectively (Fisher’s exact test). **d** The bubble chart shows the overlap significance of the three gene lists and the marker genes of each cluster in the domestic yak lung (*n* = 5 biologically independent samples) (Fisher’s exact test). SVgene: genes harboring high-FST SVs; DEGs: differentially expressed genes between the domestic yak and the taurine cattle lung; SVDEGs: DEGs harboring high-FST SVs (Note: significance, *P* < 0.05; Confidence interval = 95%; Df = 1; two-sided). Source data are provided as a Source Data file.

We then constructed plausible scenarios of causal relationships between genomic variants and development of novel cell clusters. We examined the enrichment of genes harboring high-FST SVs, DEGs, and DEGs with high-FST SVs in each sub-category of the domestic yak and taurine cattle lung cells. The T-test results showed that the cell subgroup expressed the highest proportion of marker genes carrying high FST-SV were endothelial cells in the lung of taurine cattle (Cattle6 clusters, expressing *EPAS1* and *ADGRF5*; *t* = 2.347, two-tailed, *n* = 40 and *P* = 0.0243) (Fig. 6a–d). Interestingly, the highest proportion of DEGs and DEGs carrying high-FST SVs were detected in immune cells of taurine cattle lung cells (Cattle16, expressing *THBS1* and *CXCL3*; T-test, *t* = 3.079, two-tailed, df = 40 and *P* = 0.0038 in DEGs; *t* = 3.671, two-tailed, *n* = 40 and *P* = 0.0007 in DEGs carrying high-FST SVs) and domestic yak (Yak5, expressing *THBS1* and *CXCL3*; T-test, *t* = 2.199, two-tailed, df = 42 and *P* < 0.05) (Fig. 6a–d).

Finally, we compared the proportion of SV-carrying genes, DEGs, and SV-carrying DEGs in marker genes of four cell types between domestic yak and taurine cattle (Supplementary Fig. 17). The results showed that mesenchymal cells (T-test; *P* = 0.0167; two-tailed; *t* = 3.955; df = 6) and the epithelial cells (T-test; *P* = 0.0122; two-tailed; t = 3.220; df = 10) of yak expressed more genes harboring high-FST SVs than those of cattle, however, the endothelial cells (T-test; *P* = 0.0091; two-tailed; *t* = 3.422; df = 10) of cattle expressed more DEGs than that of yak. Together, outcomes of these analyses suggested that the detected SVs were associated with differentially gene expression in different subsets of lung cells and likely had crucial roles in high altitude adaptation of yak.

## Discussion

Next-generation sequencing is a powerful tool for studying genomic variations, however, the limitation of the genome assembly using short reads makes it impossible to detect SVs thoroughly and accurately^30,31^. In contrast, Nanopore (Oxford Nanopore Technologies) has the advantage of long reads and has been proven to be effective in solving complex genomic structures^31,32^. In addition, the application of Hi-C technology provides a complementary approach for genome assembly from scratch. In the present study, with the aid of Nanopore sequencing and Hi-C data, two long-read yak genome assemblies of wild and domestic yak were constructed and released.

Although a few yak genomes have been assembled previously compared to the assembly quality of cattle^22^, cattle-yak hybrid^51^, water buffalo^52^, goat^53^, and pig genomes^54^, the quality of yak genomes needs to be improved. In this study, the lengths of contig and scaffold N50 of wild yak were 38.28 Mb and 103.90 Mb while the domestic yak assembly reached to 44.91 Mb and 104.02 Mb, respectively. The N50 estimates of these two new genomes were comparable to those of the taurine cattle genome (ARS-UCD1.2, the contig N50 at 25.89 Mb, and the scaffold N50 at 103 Mb) but better than those of existing reference genomes of wild yak (the contig N50 was 63.2 kb and the scaffold N50 was 16.3 Mb) and domestic yak (BosGru_PB_v1.0, the contig N50 at 14.74 Mb and the scaffold N50 at 101.61 Mb)^7,11^. Verified by both BUSCO and CEGMA, the integrities of these two genomes were of high quality, therefore this work will facilitate future investigation of yak genetics and genomics.

Previous studies have identified some candidate genes related to high altitude adaptation of yak, such as positively selected genes of *ADAM17*, *ARG2*, and *MMP3* by the branch-site likelihood ratio test^6^. Using a comparative transcriptomic analysis approach, it was uncovered that the heart and lung tissues exhibited the largest differences in gene expression profiles among the four organs examined (heart, liver, kidney, and lung) between yak and taurine cattle^55^. Based on second-generation short-read data, these studies provided important information on identifying candidate genes, however, the lack of high-quality genomes has hindered the efforts for assessing the impact of SVs in the yak genomes. SVs can affect the traits of animals, such as white or white-spotted pigs^56^ and white band in taurine cattle^57^, and thus provide an extensive source of genetic variations for identifying candidate genes in important biological processes. A recent study performed SV detection and comparison between domestic yak and wild yak^10^, and uncovered that 3680 SVs were under artificial selection. Importantly, more than 700 genes carrying high-FST SVs were involved in nervous system development, neuron differentiation, and behavior, suggesting possible roles of the SVs in yak domestication. Although the present study also annotated the SVs in domestic yak and wild yak, we did not describe the additional SVs in yak domestication. The focus of this work was to identify a list of candidate genes carrying high FST SVs by screening genomes of taurine cattle and yak and to further illustrate the potential roles of these genes in yak adaptation. We discovered different types of the SVs including deletions, insertions, duplications, and inversions. A limitation was that we could not select enough number of translocations for detailed analysis using current approach, although such variants were proven to be crucial for regulating gene expression and determining phenotype. For example, white-sidedness in cattle is due to serial translocation events involving KIT locus^57^. The current work primarily examined and described the SVs in exonic regions that affect gene expression, and noncoding SVs may have important consequences on influencing the expression of nearby genes and causes phenotypic variation^58^. These genes may have played crucial roles in directing the development of hypoxia tolerance at the cellular and physiological levels.

Single cell RNA-seq analyses revealed the cell components and developmental trajectories of lungs in humans, mice, and other animals. Single-cell RNA-seq data from neonatal mouse lungs suggested distinct populations of epithelial, endothelial, mesenchymal, and immune cells^41^. Single-cell map of human lungs identified 17 molecular types that either have been gained or lost during evolution or displayed substantially altered expression profiles^59^. The scRNA-seq of adult mammalian lungs, including humans, mouse, rats and pigs, revealed alveolar type I cells to play a major role in the regulation of tissue homeostasis^60^. We constructed the lung cell map of ruminants and found a unique endothelial cell population in the domestic yak lung tissue. Based on the single-cell transcriptomic and histological data, we provided candidate genes that may be related to the development of unique structure of domestic yak, including more elastic fibers and medial thickness of micro-vessels.

In summary, we present two high-quality chromosome-level genomes of wild and domestic yak. We also provided the genome-wide catalogue of SVs in yak and the single-cell transcriptomic profiles of the bovine lung tissues. These high-quality genomes and single-cell RNA-seq data serve an important source for future research on bovine species.

## Methods

### Animals

Genomic DNAs were extracted using a standard phenol-chloroform method^8^ from the blood of an adult male wild yak and an adult male domestic yak that lived above 4200 m in Qumalai County of Qinghai Province, China. Animal experiments were approved by the Animal Ethics and Welfare Committee at Northwest Institute of Plateau Biology, Chinese Academy of Sciences.

### Genome sequencing

Nanopore**:** After assessing the quality of the DNA, we constructed a Nanopore library with an insert size of 20 kb and utilized the PromethION platform to perform long-read sequencing. Hi-C: the wild yak and domestic yak blood samples were fixed in 37% formaldehyde solution. The nuclear chromatin was obtained from the fixed samples and digested using a 4-cutter restriction enzyme MboI (New England Biolabs, USA). The overhangs resulting from digestion were blunted by biotin-14-dCTP (Invitrogen, USA) and blunt-end ligation of the cross-linked fragments. Then, the genomic DNA was extracted by using the phenol-chloroform method. Biotin was removed from non-ligated fragment ends using T4 DNA polymerase (Thermo Scientific, USA). Ends of sheared fragments by sonication were repaired by the mixture of T4 DNA polymerase (Thermo Scientific, USA), T4 polynucleotide kinase (Thermo Scientific, USA), and Klenow DNA polymerase (Invitrogen, USA). Biotin-labeled Hi-C samples were specifically enriched by using streptavidin C1 magnetic beads (Invitrogen, USA). After adding A-tails to the fragment ends and following ligation by the illumina paired-end (PE) sequencing adapters, Hi-C sequencing libraries were amplified by PCR and sequenced on Illumina PE150. Illumina: The Sequencing libraries were generated using Truseq Nano DNA HT Sample preparation Kit (Illumina USA) following manufacturer’s recommendations and sequenced by Illumina HiSeq4000 platform and 150 bp paired-end reads were generated with insert size around 350 bp.

### Estimation of genome size

The k-mer algorithm was applied to evaluate the domestic yak and wild yak genome size^61^. The 17 k-mer and 171.32 Gb next-generation sequencing reads were utilized for domestic yak, while 21 k-mer and 343.13 Gb next-generation sequencing reads for wild yak.

### Genome assembly

Firstly, The two genomes were assembled directly with raw reads (Nanopore) using the long-read assembler wtdbg2^28^ (v2.5) with special parameters (domestic yak: -k 0 -p 21 -K 1000.049988 -A -S 4.000000 -s 0.070000 -g 0 -X 50.000000 -e 3 -L 0 and wild yak: -k 0 -p 21 -K 1000.049988 -A -S 4.000000 -s 0.050000 -g 0 -X 50.000000 -e 3 -L 0). Then, the assembled genome was corrected by aligning subreads using the Racon^62^ (v1.3.1) with the default parameters. Secondly, Pilon (v1.22) was used to polish the resulting assembly with 150 bp paired-end reads from the Illumina platform with special parameters (-Xmx300G–diploid–threads 20)^63^. Thirdly, The chromosome level genome were produced through Hi-C data and all-Hi-C^64^ (v0.9.8) software with the default parameters. The completeness of the assembly was evaluated by BUSCO genes and CEGMA genes. Genome quality was assessed by Merqury (v1.3) with special parameters (*k* = 21).

### Genome annotation

Repeat annotation: De novo and homology approaches were combined to identify repetitive sequences in the wild and domestic genomes. Firstly, we constructed a de novo repeat library using RepeatModeler^65^ (v2.0.1) with the default settings. Secondly, RepeatMasker^29^ (v4.1.0) was run on the wild and domestic yak genomes using the de novo library. RepeatMasker^29^ and its in-house scripts (RepeatProteinMask) was also run against the RepBase for homologous repeat identification.

Gene structure annotation: Homology-based prediction, ab initio prediction, and RNA-seq assisted prediction were used to annotate all potential genes. (1) Homolog prediction: Sequences of homologous proteins of six related ruminant species, including buffalo (*Bubalus_bubalis*), cattle (*Bos_taurus*), sheep (*Ovis_aries*), goat (*Capra_hircus*), European bison (*Bison_bonasus*) and reindeer (*Rangifer_tarandus*), were downloaded from Ensembl/NCBI/Gigadb database. These protein sequences were aligned to the wild and domestic yak genomes using TblastN^66^ (v2.2.26; E-value ≤ 1e − 5) based on the default parameters. The matching proteins were then aligned to the homologous genome sequences for accurately spliced alignments using GeneWise^67^ (v2.4.1) package to predict gene structure in each protein region. (2) Ab initio prediction: Augustus^68^ (v3.3.2), Geneid^69^ (v1.4), Genescan (v1.0), GlimmerHMM^70^ (v3.0.4), and Snap (v2013.11.29) were used in our automated gene prediction pipeline. (3) RNA-seq data: After QC and filtering, reads from all RNA libraries were mapped to the wild and domestic yak genome using Hisat^71^ (v2.0.4) and Stringtie^72^ (v1.3.3) was subsequently used to predict gene models with default parameters. All predicted genes from the three approaches were integrated with EVidenceModeler (EVM)^73^ (v1.1.1) to generate high-confidence gene sets.

Gene function annotation: Gene functions were assigned according to the best match by aligning the protein sequences using Blastp^74^ (v2.2.26, E-value <10^−5^) to protein databases including the SwissProt, Nr, Pfam, KEGG, and InterPro. The best hits were used to assign homology-based gene functions. Functional classification based on GO categories and InterPro entries was achieved using the InterProScan (v5.35).

Non-coding RNA annotation: The tRNAs were predicted using the program tRNAscan-SE (v1.4). Because rRNAs are highly conserved, we choose relative species’ rRNA sequence as references, including buffalo (*Bubalus_bubalis*), cattle (*Bos_taurus*), sheep (*Ovis_aries*), goat (*Capra_hircus*), European bison (*Bison_bonasus*) and reindeer (*Rangifer_tarandus*), predict rRNA sequences using Blast. Other ncRNAs, including miRNAs and snRNAs were identified by searching against the Rfam database with default parameters using the infernal software (v1.1.2).

### Structural variant analysis

The genome of a female taurine cattle (ARS-UCD1.2) and the Y chromosome of other male taurine cattle (Btau_4.6.1) were downloaded and merged into the taurine cattle genome file. For Nanopore sequenced data, we aligned each sample to ARS-UCD1.2 using NGMLR (v0.2.7) with the option ‘–x ont’, while without ‘the option ‘–x ont’for Pacbio sequenced data. Alignments were sorted and indexed by samtools (v1.8). The program cuteSV (v1.0.11) was used to call SVs for each individual with important parameters 1 (–max_cluster_bias_INS 100–diff_ratio_merging_INS 0.3–max_cluster_bias_DEL 100–diff_ratio_merging_DEL 0.3) for Nanopore data and important parameters 2 (–max_cluster_bias_INS 100–diff_ratio_merging_INS 0.3–max_cluster_bias_DEL 200–diff_ratio_merging_DEL 0.5) for pacbio data. We merged all VCF files to obtain a total SV data set by SURVIVOR (v1.0.7) with parameters ‘50 1 1 −1 −1 −1’. cuteSV with option ‘–genotype’ was used again to perform genotyping for each sample at all SV breakpoints based on the merged VCF file and all mapping results. Then we performed a filtration to remove unreliable SVs using vcftools (v0.1.17) with parameters (–max-missing 0.5–mac 3–minQ 30). The python script was used to calculate the number of SVs on each chromosome, the proportion of different SVs, the size distribution of SVs, the chromosome distribution of different SVs, and the sample distribution of different SVs. The filtered SVs were annotated used VEP (Web version).

We verified the selected SVs through PCR. DNAs were extracted from testes of three male taurine cattle and three male domestic yak using a standard phenol-chloroform method^8^ in Xining County of Qinghai Province, China. The regions of selected SVs were amplified using the primers (Supplementary Table 26). The PCR program consisted of an initial denaturing step at 95 °C for 4 min, 35 amplification cycles (95 °C for 55 s, 60 °C for 55 s, and 72 °C for 55 s), and a final extension at 72 °C for 5 min in a thermocycler (Eppendorf).

### Identification and enrichment analysis of differentially expressed genes

The transcriptomic data of yak and taurine cattle were retrieved (Supplementary Table 23). The clean data were aligned to the reference genome (ARS-UCD1.2) using hisat2 (v2.2.1). After alignments were sorted and indexed by samtools, counts are obtained through the featureCounts program with parameters (-p -t exon -g transcript_id). DESeq2^75^ package (v1.30.1) was used to analyze the differentially expressed transcript (DET) between the taurine cattle and yak lungs. The R script is used to calculate the *p*-value of each transcript by the t-test, and DETs are screened by *p*-value <0.05 and |log2FoldChange|> = 1. Gene ID conversion and enrichment analysis were performed through the g:Profiler site^76^. Miropeats^77^ (v2.02) was used to visualize the SVs in specific genes (*TXNDC5*, *TENM1 and ZCHHC14*). Gene expression histograms were drawn using Graphpad prism software (v8.0). The Veen diagrams of differentially expressed genes (DEGs) and the genes with SVs were drawn. The one-way ANOVA and multiple comparisons was done with the GraphPad Prism software.

### Analysis of Yak-specific gene expression

Orthofinder software (v2.5.4), domestic yak, and cattle (ARS-UCD1.2) amino acid files were used to calculate yak-specific genes^78^. Transcriptome data from yak lung were used to realign to the domestic yak reference genome using hisat2, and then the count value was calculated using the featureCounts program, and finally the normalized count value was calculated using the DEseq2 package.

### Single-cell transcriptome sequencing

The lung tissue samples were collected from 5 adult male taurine cattle in Zhangye (Gansu province, China) and 5 adult male domestic yak in Xining (Qinghai province, China). The samples were transported to the lab on ice and digested with 5 ml 1 mg/ml collagenase for 20 min until the lung tissues were digested into single cells. After adherent cells were discarded using a 40 µm cell filter, the single cell suspension was centrifuged with 400 g for 5 min and then suspended in 5 ml Dulbecco’s phosphate-buffered saline (DPBS) containing 10% FBS (DPBS + S). Red blood cells were removed using Solarbio erythrocyte lysis buffer (3:1; Beijing, China). After centrifugation, sample buffer (BD Biosciences, New Jersey, USA) was added for single cell RNA sequence determination. BD Rhapsody^TM^ Single Cell Analysis System (DOCID: 210966 Rev. 1.0 protocol) was used for single-cell cDNA synthesis. The library preparation was performed according to the BD Rhapsody^TM^ Whole Transcriptome Analysis (WTA) Amplification Kit and BD AbSeq library preparation protocol. In particular, we used three taurine cattle samples to build the library, and two taurine cattle samples for the second time. For the five yak samples, we adopted the same method to build the library. Double-ended sequencing of 150 bp was performed on the IIIumina HiSeq 2000 platform by Novogene (Beijing, China).

### Single-cell transcriptome data analysis

Quality control and analysis of the original data were performed according to BD Phapsody pipeline. STAR (v2.7.1a) was used to index the reference genome (Taurine cattle: the same as the reference genome of sv calling; Yak: the newly assembled domestic yak reference genome in this study)^79^. After quality filtering, reference gene comparison, expression quantification, and a cell-gene expression matrix file were generated following BD pipeline. The expression matrix obtained from the first and the second are integrated by CCA and then was processed by cluster analysis using Seurat toolkit^50^ (v3.2.0) with following protocols: (1) Quality control and cell filtration: Low-quality cells were filtered according to the expression of genes. The filtering standards were <300 and <500 detected (transcript count > 0) genes for the taurine cattle and yak lungs. >20% of transcript counts were mapped to mitochondrial genes for the lungs of both species; (2) Data standardization and noise reduction: The default global normalization method “LogNormalize” of Seurat toolkit was used to standardize the gene expression matrix; (3) Cell clustering: Principal Components Analysis (PCA) was performed, then the most significant principal components were evaluated, and the first 20 principal components were selected for cluster analysis. According to the clustering results, the UMAP method^40^ was used to visualize the distribution of cells in two-dimensional space, and the cells of the same cluster were represented by the same color (important parameter: dims = 1:20, check_duplicates =  FALSE); (4) Search and visualization of marker genes: Seurat toolkit was used to calculate each marker gene and its expression level in each cluster with default parameters and filter marker genes with special parameters (p_val_adj <0.05 and avg_logFC > 1). Special marker genes were displayed in violin map using Seurat toolkit and the heatmap was generated using ComplexHeatmap package^80^ (v2.6.2). (5) Differential expression analysis: Use FindMarkers function to detect differentially expressed genes in mesenchymal cells of cattle and yak with special parameters “min.pct=0.25”. (6) Functional enrichment analysis: g:Profiler web server was used to analyze the GO functional and KEGG pathway enrichments of each set of differentially and highly expressed genes. The ggplot2 package (v3.3.3) was used to draw bar charts for visualization of the results. (7) We computed the Pearson correlation coefficient between the yak and taurine cattle lungs using corr.test function in psych package (v2.0.8).

### Two-sample integrative analysis

The two expression matrices filtered in single sample analysis were used for two-sample integrative analysis. The FindIntegrationAnchors function in the Seurat toolkit was applied to identify the anchors which were qualified for the IntegrateData function in the Seurat toolkit, and the integrated data were returned to a Seurat toolkit object, which contained a new Assay (integrated), which stored the integrated expression matrix (Important parameters: dims = 1:30). The UMAP method^40^ was used for cell clustering (Important parameters: FindClusters (resolution = 0.1)); RunUMAP (reduction = “pca”, dims = 1:30). The Seurat toolkit was used to calculate each marker gene and its expression level in each cluster (important parameters: FindMarkers (default parameters); and filter (p_val_adj <0.05 and avg_logFC > 1)). The ComplexHeatmap package was used for visualization of the marker genes in a heat map. The ggplot2 package and the Seurat toolkit were used to draw a histogram and a scatter diagram.

### Integrated analysis of scRNA-seq, genes with SVs, DEGs, and DEGs with SVs

Four gene lists including (1) The genes with high-FST SVs; (2) The DEGs between the taurine cattle and yak lungs; (3) The intersection of the first two genes; and (4) The marker gene of each cluster were used for integrated analysis. The proportions of the first three gene sets in each cluster (marker gene set) were calculated and normalized. The chi-square test was done with the stats R package. The *p-*value and number were determined using ggplot2 package. The T-test were done with the GraphPad Prism software.

### Elastic fiber and Hematoxylin & Eosin (HE) staining

Lungs tissues from taurine cattle and yak were fixed in 4% paraformaldehyde and cut in serial paraffin sections (5–7 μm in thickness). Sections were processed for HE staining or elastic fiber staining. Elastic fibers were stained in aldehyde fuchsin solution for 10 min. Digital images were captured with a microscope (Nikon ELIPSE E200, Japan). All quantitative data were counted for at least three independent biological replicates. The two-tailed t-test analysis function of the GraphPad Prism software was used to statistically determine the difference between the means and the significance was set to *P* < 0.05.

### Reporting summary

Further information on research design is available in the Nature Research Reporting Summary linked to this article.

## Supplementary information

Supplementary Information

Description of Additional Supplementary Files

Supplementary Data 1

Supplementary Data 2

Supplementary Data 3

Supplementary Data 4

Supplementary Data 5

Supplementary Data 6

Supplementary Data 7

Reporting Summary

## Data Availability

We have deposited the assembled genome, raw Hic data, and raw Nanopore data of wild yak and domestic yak at Sequence Read Archive (SRA) database of National Center for Biotechnology Information (NCBI) database with accession BioProject codes: PRJNA720245 and PRJNA720246. And the lung single-cell RNA-Sequencing data of domestic yak and taurine cattle have been at the NCBI database with accession BioProject codes: PRJNA720247 and PRJNA720248. Source data for Figs. 1a, c, d, 2a, c, 3c, 4d, e, 5c, 6a–d, Supplementary Fig. 11, Supplementary Fig. 16, and Supplementary Fig. 17 are provided with this paper. Source data are provided with this paper.

## Source data

Source data

## Code availability

Scripts and codes are available at https://github.com/GaoXue96/Yak_adaptation.

## References

[1] Guo Y, Zhou Y, Shi Q, Meng X (2018). Endangered wild yak: distribution,population,impacting factors and conservation. Chin. J. Wildl..

[2] Meng Q (2017). The distribution on characteristics and populations of yak. Acta Ecologiae Anim. Domastici.

[3] Li R (2014). Novel Y-chromosome polymorphisms in Chinese domestic yak. Anim. Genet..

[4] Wang Z (2011). Domestication relaxed selective constraints on the yak mitochondrial genome. Mol. Biol. Evol..

[5] Zhang K, Lenstra JA, Zhang S, Liu W, Liu J (2020). Evolution and domestication of the Bovini species. Anim. Genet..

[6] Qiu Q (2012). The yak genome and adaptation to life at high altitude. Nat. Genet..

[7] Liu Y (2020). The sequence and de novo assembly of the wild yak genome. Sci. Data.

[8] Guo S (2006). Origin of mitochondrial DNA diversity of domestic yaks. BMC Evol. Biol..

[9] Wang ZF (2010). Phylogeographical analyses of domestic and wild yaks based on mitochondrial DNA: new data and reappraisal. J. Biogeogr..

[10] Zhang S (2021). Structural variants selected during yak domestication inferred from long-read whole-genome sequencing. Mol. Biol. Evol..

[11] Ji, Q. M. et al. A chromosome-scale reference genome and genome-wide genetic variations elucidate adaptation in yak. *Mol. Ecol. Resour*. **21**, 201–211 (2020). 10.1111/1755-0998.13236 PMC7754329 32745324

[12] Lan D (2018). Genetic diversity, molecular phylogeny, and selection evidence of jinchuan yak revealed by whole-genome resequencing. G3.

[13] Wang K (2014). Genome-wide variation within and between wild and domestic yak. Mol. Ecol. Resour..

[14] Qiu Q (2015). Yak whole-genome resequencing reveals domestication signatures and prehistoric population expansions. Nat. Commun..

[15] Zhang X (2016). Genome-wide patterns of copy number variation in the Chinese yak genome. BMC Genom..

[16] Medugorac I (2017). Whole-genome analysis of introgressive hybridization and characterization of the bovine legacy of Mongolian yaks. Nat. Genet..

[17] Xie X (2018). Accumulation of deleterious mutations in the domestic yak genome. Anim. Genet..

[18] Lan D (2020). Population genome of the newly discovered Jinchuan yak to understand its adaptive evolution in extreme environments and generation mechanism of the multirib trait. Integr. Zool..

[19] Qin P (2021). Pan-genome analysis of 33 genetically diverse rice accessions reveals hidden genomic variations. Cell.

[20] Sudmant PH (2015). An integrated map of structural variation in 2504 human genomes. Nature.

[21] Abel HJ (2020). Mapping and characterization of structural variation in 17,795 human genomes. Nature.

[22] Rosen BD (2020). De novo assembly of the cattle reference genome with single-molecule sequencing. Gigascience.

[23] Chen Q, Feng X, Jiang S (2006). Structural study on plateau adaptability of yak lung. Sci. Agric. Sin..

[24] Xiao W, Tian Y, Ge C (1997). Study on slaughter performance of Zhongdian yak. J. Yunnan Anim. Vet. Sci..

[25] Yang C (2017). Research progress on adaptation on the histology and anatomy in Yak(Bos grunniens) in Qinghai-Tibetan Plateau. Chin. J. Anim. Sci..

[26] Qi XY, Ma L, Yang SL, Kong XY, Yan DW (2017). Study on the blood physiological representation for hypoxia adaptation in yaks and Tibetan cattle. Hlongjiang Anim. Sci. Vet. Med..

[27] Qi X (2019). The transcriptomic landscape of yaks reveals molecular pathways for high altitude adaptation. Genome Biol. Evol..

[28] Ruan J, Li H (2020). Fast and accurate long-read assembly with wtdbg2. Nat. Methods.

[29] Tarailo-Graovac, M. & Chen, N. *Using RepeatMasker to identify repetitive elements in genomic sequences*, Unit 4.10, 1–14 (2009). 10.1002/0471250953.bi0410s25 19274634

[30] Shi L (2016). Long-read sequencing and de novo assembly of a Chinese genome. Nat. Commun..

[31] Norris AL, Workman RE, Fan Y, Eshleman JR, Timp W (2016). Nanopore sequencing detects structural variants in cancer. Cancer Biol. Ther..

[32] Alonge M (2020). Major impacts of widespread structural variation on gene expression and crop improvement in tomato. Cell.

[33] Sedlazeck FJ (2018). Accurate detection of complex structural variations using single-molecule sequencing. Nat. Methods.

[34] Jiang T (2020). Long-read-based human genomic structural variation detection with cuteSV. Genome Biol..

[35] Shiratsuki S (2016). Low oxygen level increases proliferation and metabolic changes in bovine granulosa cells. Mol. Cell Endocrinol..

[36] Welch JJ (2004). Global regulation of erythroid gene expression by transcription factor GATA-1. Blood.

[37] Ueda K, Xu J, Morimoto H, Kawabe A, Imaoka S (2008). MafG controls the hypoxic response of cells by accumulating HIF-1α in the nuclei. Febs Lett..

[38] Li XC (2016). KLF5 mediates vascular remodeling via HIF-1 alpha in hypoxic pulmonary hypertension. Am. J. Physiol.-Lung C..

[39] Volpe MV (2013). Regulatory interactions between androgens, Hoxb5, and TGFβ signaling in murine lung development. Biomed. Res. Int..

[40] Becht E (2018). Dimensionality reduction for visualizing single-cell data using UMAP. Nat. Biotechnol..

[41] Guo, M. et al. Single cell RNA analysis identifies cellular heterogeneity and adaptive responses of the lung at birth. *Nat. Commun.* **10**, 37 (2019). 10.1038/s41467-018-07770-1 PMC6318311 30604742

[42] Hanaoka M (2012). Genetic variants in EPAS1 contribute to adaptation to high-altitude hypoxia in Sherpas. PLoS One.

[43] Peng YR (2019). Molecular classification and comparative taxonomics of foveal and peripheral. Cells Primate Retin. Cell.

[44] Tosches MA (2018). Evolution of pallium, hippocampus, and cortical cell types revealed by single-cell transcriptomics in reptiles. Science.

[45] Lee TH (2020). Fibroblast-enriched endoplasmic reticulum protein TXNDC5 promotes pulmonary fibrosis by augmenting TGFbeta signaling through TGFBR1 stabilization. Nat. Commun..

[46] Alkelai A (2016). A role for TENM1 mutations in congenital general anosmia. Clin. Genet..

[47] Handrigan GR (2013). Deletions in 16q24.2 are associated with autism spectrum disorder, intellectual disability and congenital renal malformation. J. Med. Genet..

[48] Wagenseil JE, Mecham RP (2007). New insights into elastic fiber assembly. Birth Defects Res C. Embryo Today.

[49] Butler A, Hoffman P, Smibert P, Papalexi E, Satija R (2018). Integrating single-cell transcriptomic data across different conditions, technologies, and species. Nat. Biotechnol..

[50] Stuart T (2019). Comprehensive Integration of Single-. Cell Data. Cell.

[51] Rice, E. S. et al. Continuous chromosome-scale haplotypes assembled from a single interspecies F1 hybrid of yak and cattle. *Gigascience* **9**, giaa029 (2020). 10.1093/gigascience/giaa029 PMC7118895 32242610

[52] Low WY (2019). Chromosome-level assembly of the water buffalo genome surpasses human and goat genomes in sequence contiguity. Nat. Commun..

[53] Bickhart DM (2017). Single-molecule sequencing and chromatin conformation capture enable de novo reference assembly of the domestic goat genome. Nat. Genet..

[54] Warr A (2020). An improved pig reference genome sequence to enable pig genetics and genomics research. Gigascience.

[55] Wang K (2016). Different gene expressions between cattle and yak provide insights into high-altitude adaptation. Anim. Genet..

[56] Rubin CJ (2012). Strong signatures of selection in the domestic pig genome. Proc. Natl Acad. Sci..

[57] Durkin K (2012). Serial translocation by means of circular intermediates underlies colour sidedness in cattle. Nature.

[58] Scott, A. J., Chiang, C. & Hall, I. M. Structural variants are a major source of gene expression differences in humans and often affect multiple nearby genes. *Genome Res.* **31**, 2249–2257 (2021). 10.1101/gr.275488.121 PMC8647827 34544830

[59] Travaglini KJ (2020). A molecular cell atlas of the human lung from single-cell RNA sequencing. Nature.

[60] Raredon MSB (2019). Single-cell connectomic analysis of adult mammalian lungs. Sci. Adv..

[61] Shao Y (2020). Genome and single-cell RNA-sequencing of the earthworm Eisenia andrei identifies cellular mechanisms underlying regeneration. Nat. Commun..

[62] Vaser R, Sovic I, Nagarajan N, Sikic M (2017). Fast and accurate de novo genome assembly from long uncorrected reads. Genome Res..

[63] Walker BJ (2014). Pilon: an integrated tool for comprehensive microbial variant detection and genome assembly improvement. PLoS One.

[64] Zhang X, Zhang S, Zhao Q, Ming R, Tang H (2019). Assembly of allele-aware, chromosomal-scale autopolyploid genomes based on Hi-C data. Nat. Plants.

[65] Flynn JM (2020). RepeatModeler2 for automated genomic discovery of transposable element families. Proc. Natl Acad. Sci. USA.

[66] Gertz EM, Yu YK, Agarwala R, Schaffer AA, Altschul SF (2006). Composition-based statistics and translated nucleotide searches: improving the TBLASTN module of BLAST. BMC Biol..

[67] Birney E, Clamp M, Durbin R (2004). GeneWise and genomewise. Genome Res.

[68] Hoff KJ, Stanke M (2019). Predicting genes in single genomes with AUGUSTUS. Curr. Protoc. Bioinform..

[69] Alioto T, Blanco E, Parra G, R G (2018). Using geneid to Identify Genes. Curr. Protoc. Bioinform..

[70] Majoros WH, Pertea M, Salzberg SL (2004). TigrScan and GlimmerHMM: two open source ab initio eukaryotic gene-finders. Bioinformatics.

[71] Kim D, Langmead B, Salzberg SL (2015). HISAT: a fast spliced aligner with low memory requirements. Nat. Methods.

[72] Pertea M, Kim D, Pertea GM, Leek JT, Salzberg SL (2016). Transcript-level expression analysis of RNA-seq experiments with HISAT, StringTie and Ballgown. Nat. Protoc..

[73] Haas BJ (2008). Automated eukaryotic gene structure annotation using EVidenceModeler and the Program to Assemble Spliced Alignments. Genome Biol..

[74] Camacho C (2009). BLAST+: architecture and applications. BMC Bioinform..

[75] Love MI, Huber W, Anders S (2014). Moderated estimation of fold change and dispersion for RNA-seq data with DESeq2. Genome Biol..

[76] Raudvere U (2019). g:Profiler: a web server for functional enrichment analysis and conversions of gene lists (2019 update). Nucleic Acids Res..

[77] Parsons JD (1995). Miropeats: graphical DNA sequence comparisons. Comput. Appl. Biosci..

[78] Emms DM, Kelly S (2019). OrthoFinder: phylogenetic orthology inference for comparative genomics. Genome Biol..

[79] Dobin A (2013). STAR: ultrafast universal RNA-seq aligner. Bioinformatics.

[80] Gu Z, Eils R, Schlesner M (2016). Complex heatmaps reveal patterns and correlations in multidimensional genomic data. Bioinformatics.

