# Supplementary material for: Long read genome assemblies complemented by single cell RNA-sequencing reveal genetic and cellular mechanisms underlying the adaptive evolution of yak
Source: Nat Commun. 2022 Sep 6;13:4887. doi: 10.1038/s41467-022-32164-9 (PMC9448747; doi:10.1038/s41467-022-32164-9)
Supplement: Supplementary file 1 — Supplementary Information [file 41467_2022_32164_MOESM1_ESM.pdf]

Long read genome assemblies complemented by single cell RNA-sequencing reveal genetic and cellular mechanisms underlying the adaptive evolution of yak

Gao, Wang, et al.

This PDF file includes:

1) Supplementary Fig. 1-17
2) Supplementary Tables 1-26

1 Animals

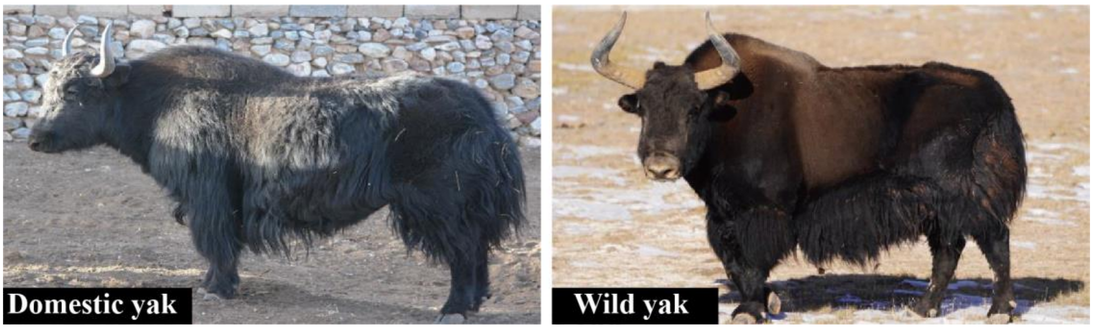

Supplementary Fig. 1. Representative images of a domestic yak and a wild yak.

2 Annotation

2.1 Repetitive sequence annotation

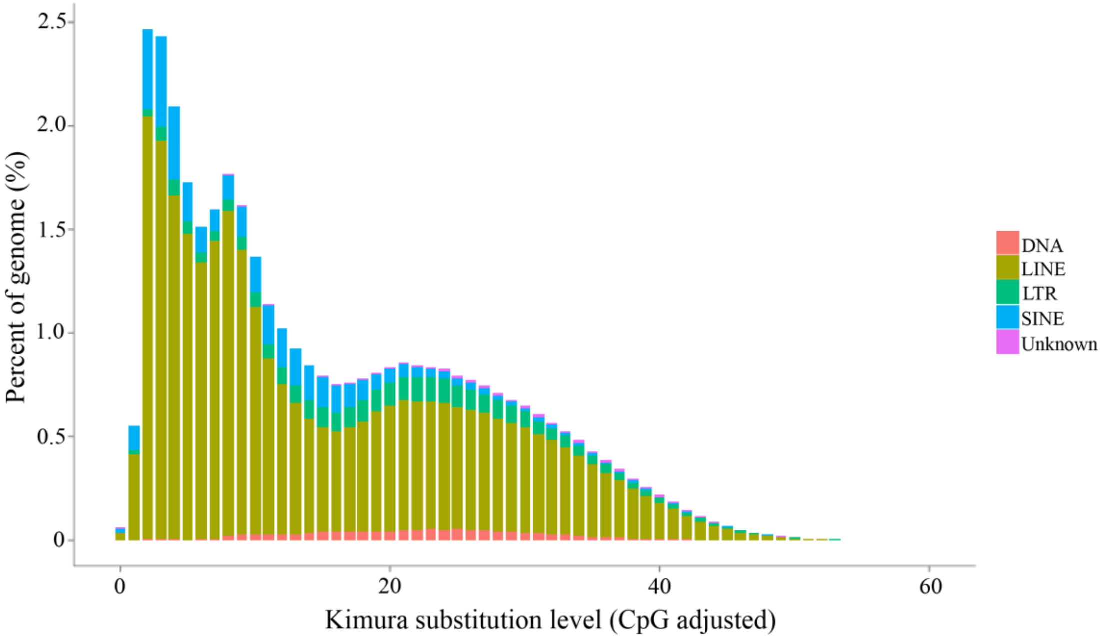

Supplementary Fig. 2. Distribution map of genomic TE sequence divergence in domestic yak. The abscissa is the degree of divergence between the TE sequence annotated in the domestic yak genome and the corresponding sequence in the Repbase. The ordinate is the percentage of the TE sequence in the genome at that divergence, and different colors indicate various TEs.

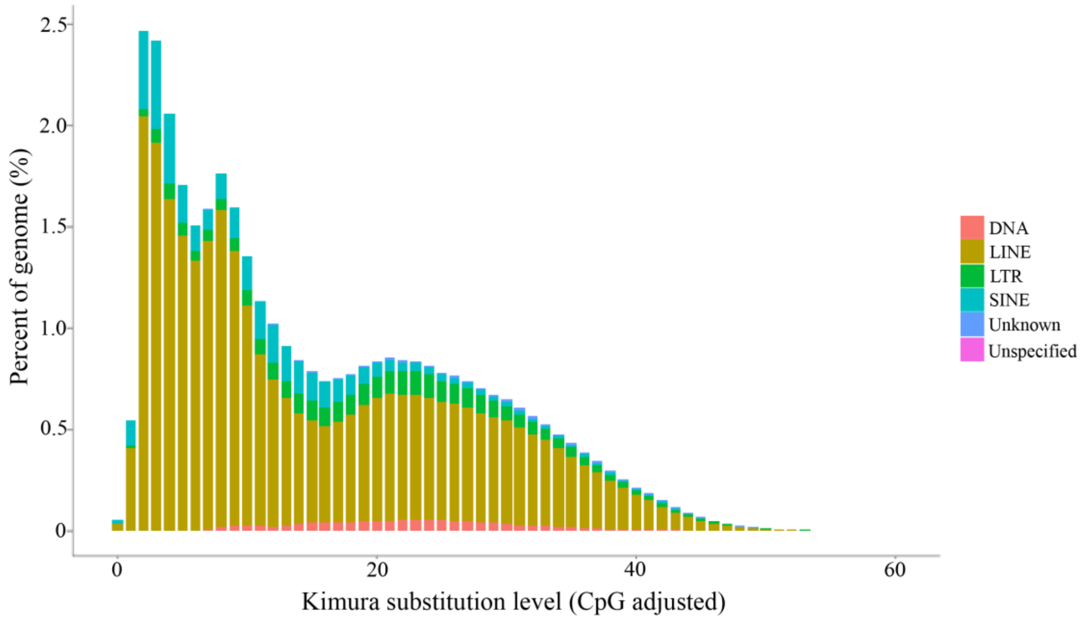

Supplementary Fig. 3. Distribution map of genomic TE sequence divergence in wild yak. The abscissa is the degree of divergence between the TE sequence annotated in the wild yak genome and the corresponding sequence in the Repbase; the ordinate is the percentage of the TE sequence in the genome at that divergence, and different colors indicate various TEs.

2.2 Gene structure annotation

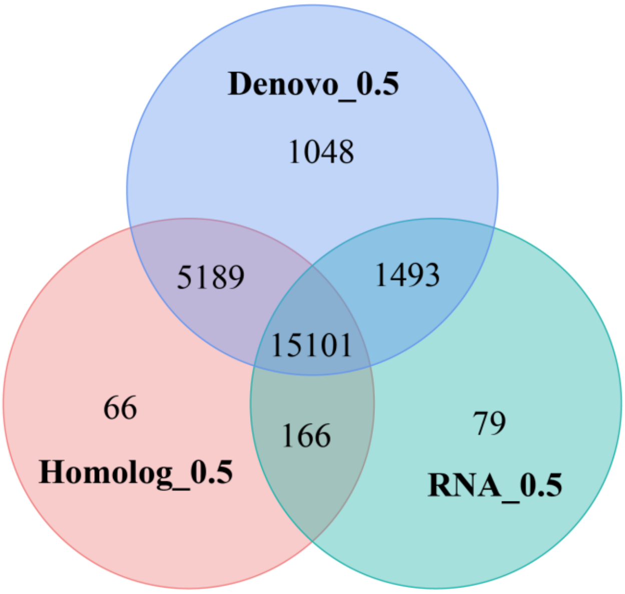

Supplementary Fig. 4. Statistics of genome gene set of domestic yak. Note: Denovo, EVM integrates genes supported by De novo prediction; Homolog, genes supported by homology prediction during EVM integration; RNA, genes supported by RNA-seq during EVM integration; each evidence support is based on gene overlap greater than 50% Standard; the number indicates the number of genes.

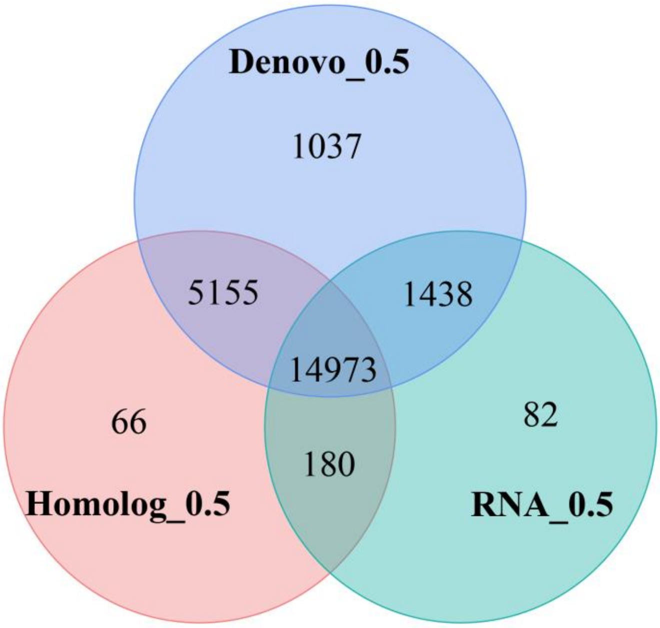

Supplementary Fig. 5. Statistics of genome gene set of wild yak. Note: Denovo, EVM integrates genes supported by De novo prediction; Homolog, genes supported by homology prediction during EVM integration; RNA, genes supported by RNA-seq during EVM integration; each evidence support is based on gene overlap greater than 50% Standard; the number indicates the number of genes.

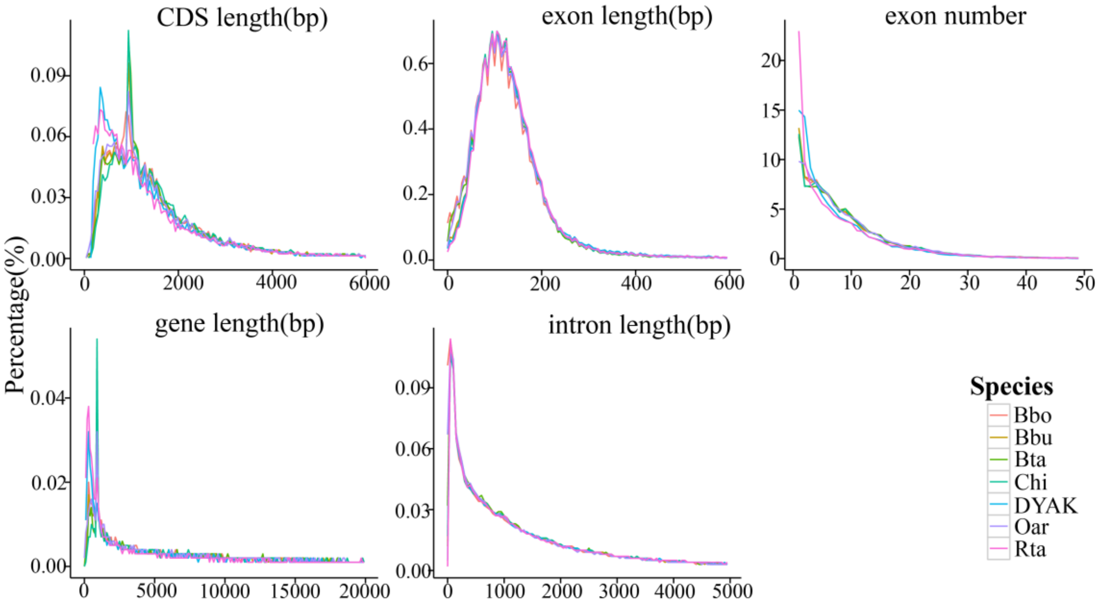

Supplementary Fig. 6. Comparative diagrams of components between domestic yak and related species. Note: Bbu(*Bubalus_bubalis*); Bta(*Bos_taurus*); Oar(*Ovis_aries*); Chi(*Capra_hircus*); Bbo(*Bison_bonasus*); Rta(*Rangifer_tarandus*); DGYAK(*Bos grunniens*, this study).

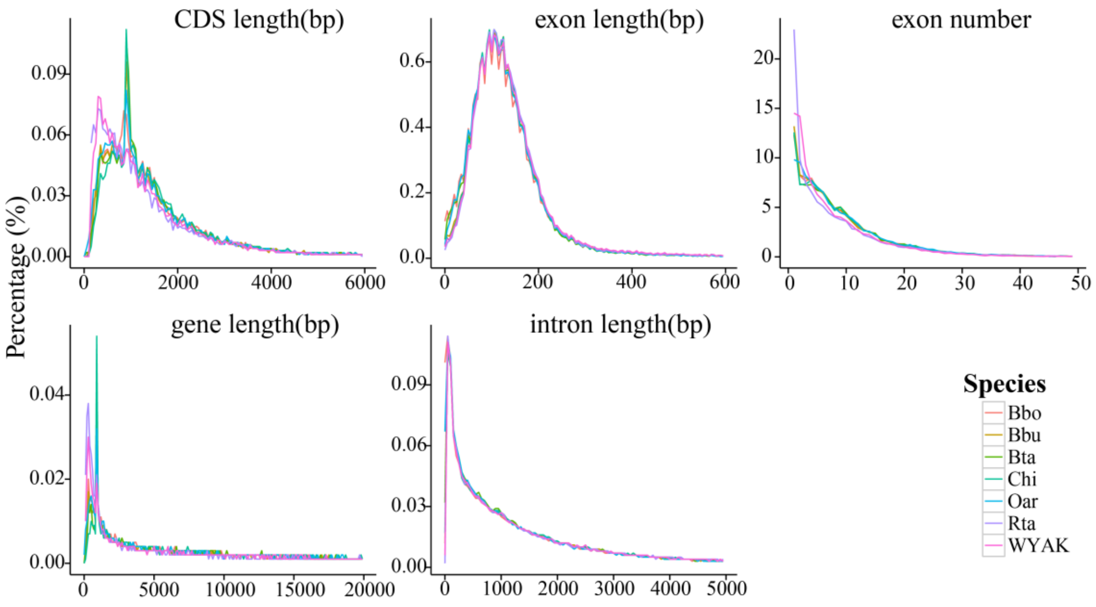

Supplementary Fig. 7. Comparative diagrams of components between wild yak and related species. Note: Bbu(*Bubalus_bubalis*); Bta(*Bos_taurus*); Oar(*Ovis_aries*); Chi(*Capra_hircus*); Bbo(*Bison_bonasus*); Rta(*Rangifer_tarandus*); WYAK(*Bos mutus,* this study).

2.3 Gene function annotation

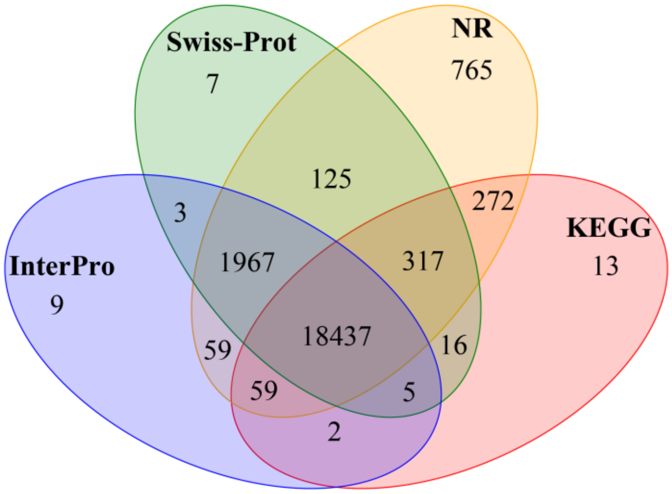

Supplementary Fig. 8. Statistical results of gene function annotation in domestic yak. Note: Align the known protein library with the protein sequence obtained by gene structure prediction. Each color represents a different database.

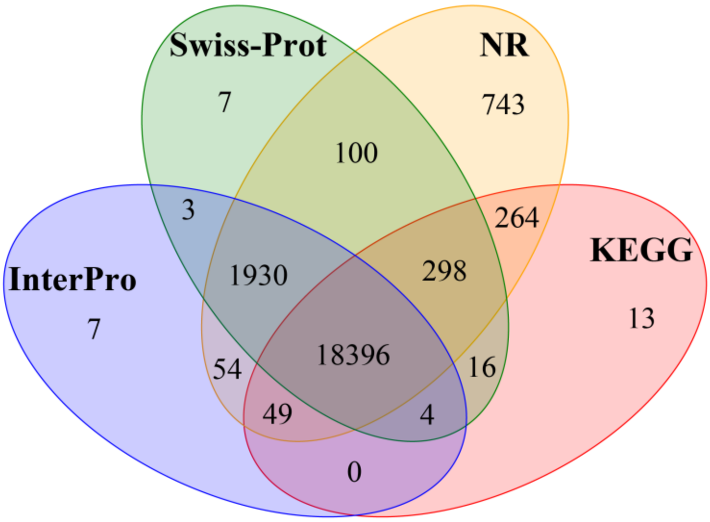

Supplementary Fig. 9. Statistical results of gene function annotation in domestic yak. Note: Align the known protein library with the protein sequence obtained by gene structure prediction. Each color represents a different database.

3 Structural variations

3.1 SVs visualization

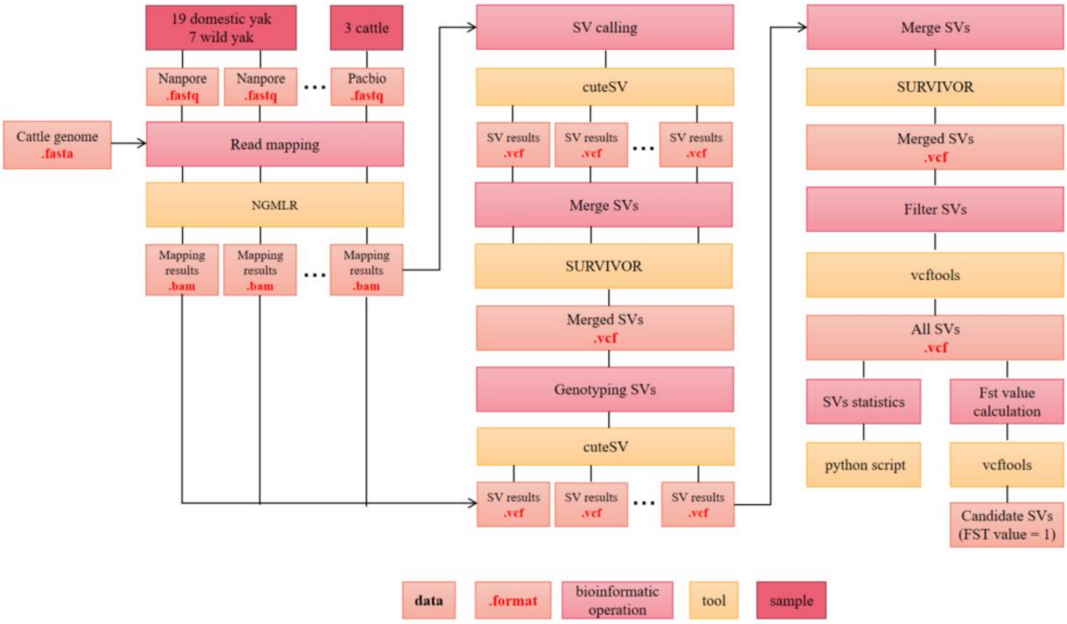

Supplementary Fig. 10. Work flow for SV detection based on long-read sequencing.

3.2 SVs statistics

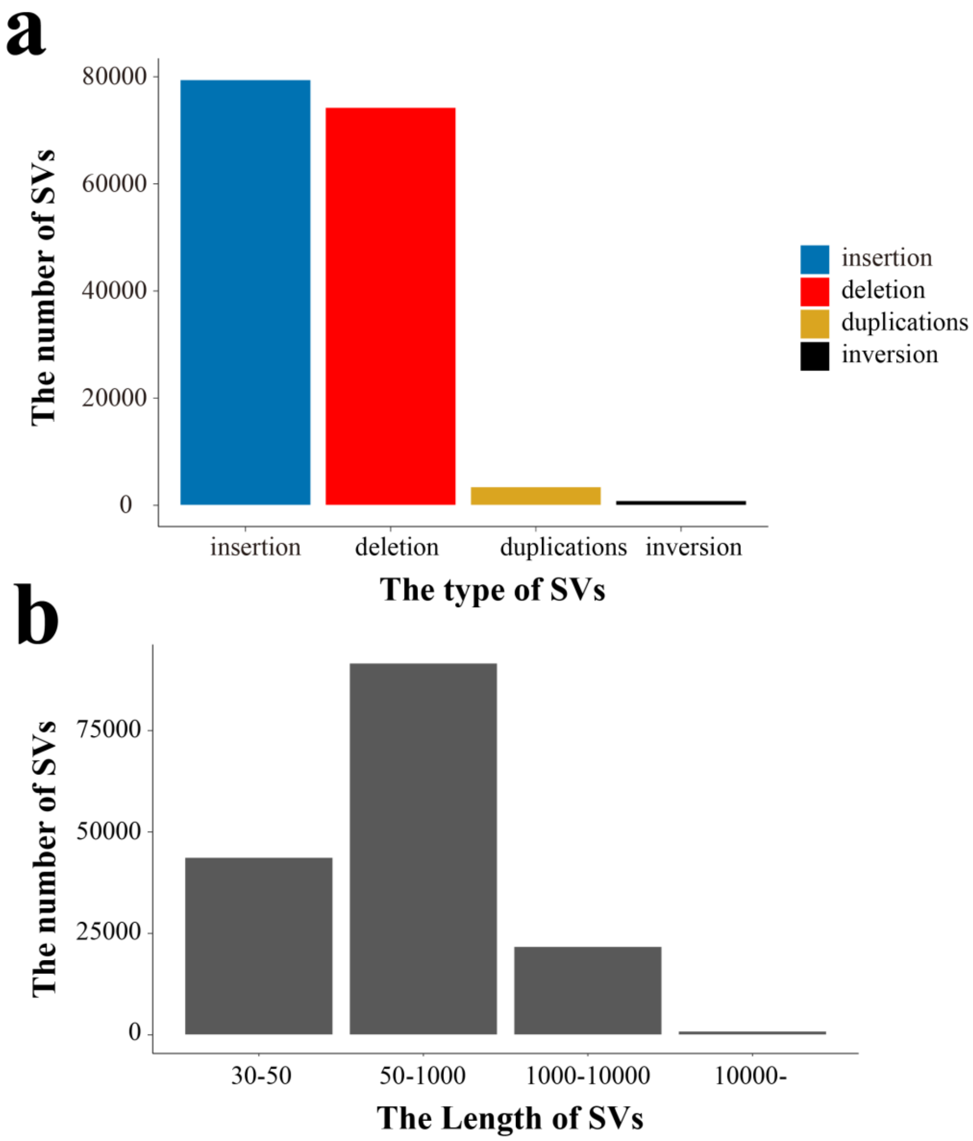

Supplementary Fig. 11. The statistics results of SVs. a, The histogram shows the number of SVs with different types. **b,** The histogram shows the number of SVs with different length. Source data are provided as a Source Data file.

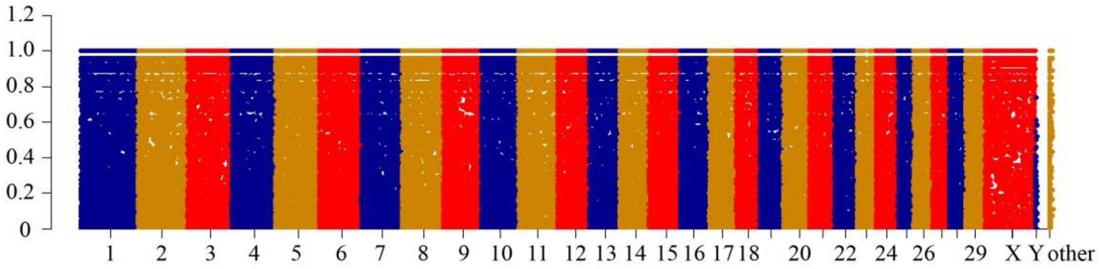

Supplementary Fig. 12. Manhattan plot of the FST values of all chromosome calculated (left) between the taurine cattle and yak.

4. Single sample analysis of scRNA-seq

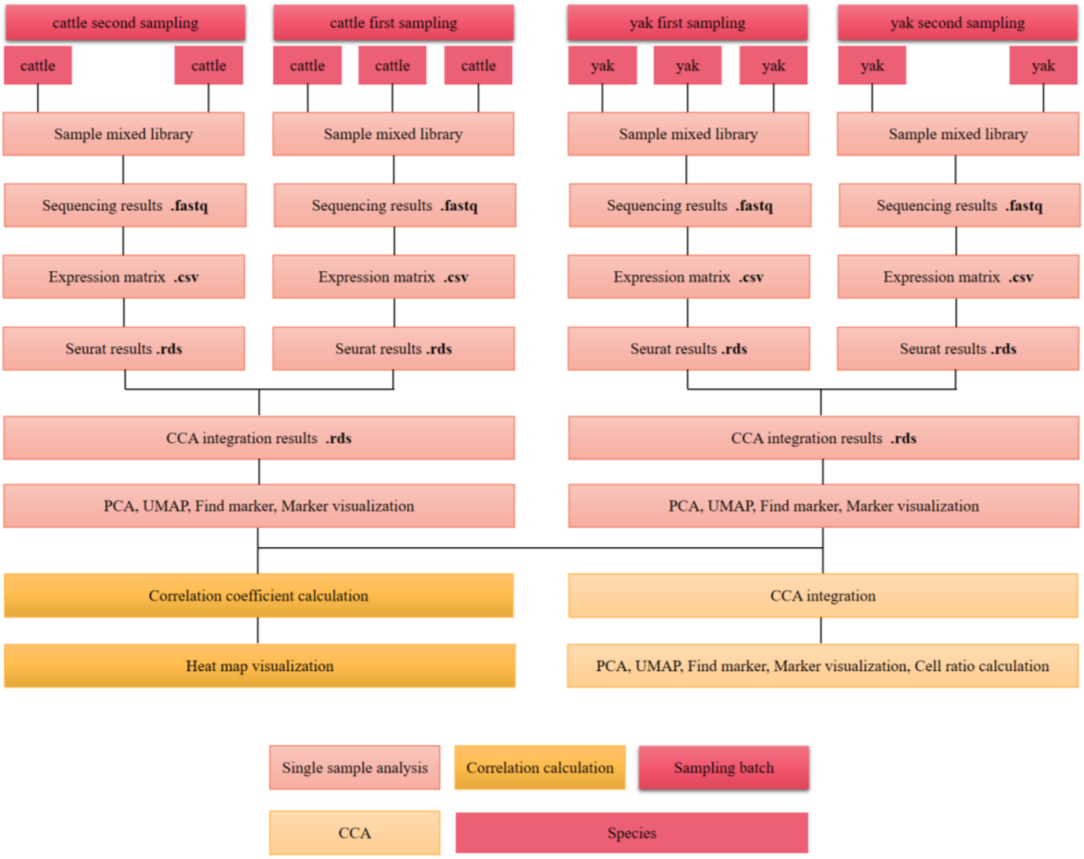

Supplementary Fig. 13. Work flow for single cell RNA-seq analysis of lung tissues from taurine cattles and yaks.

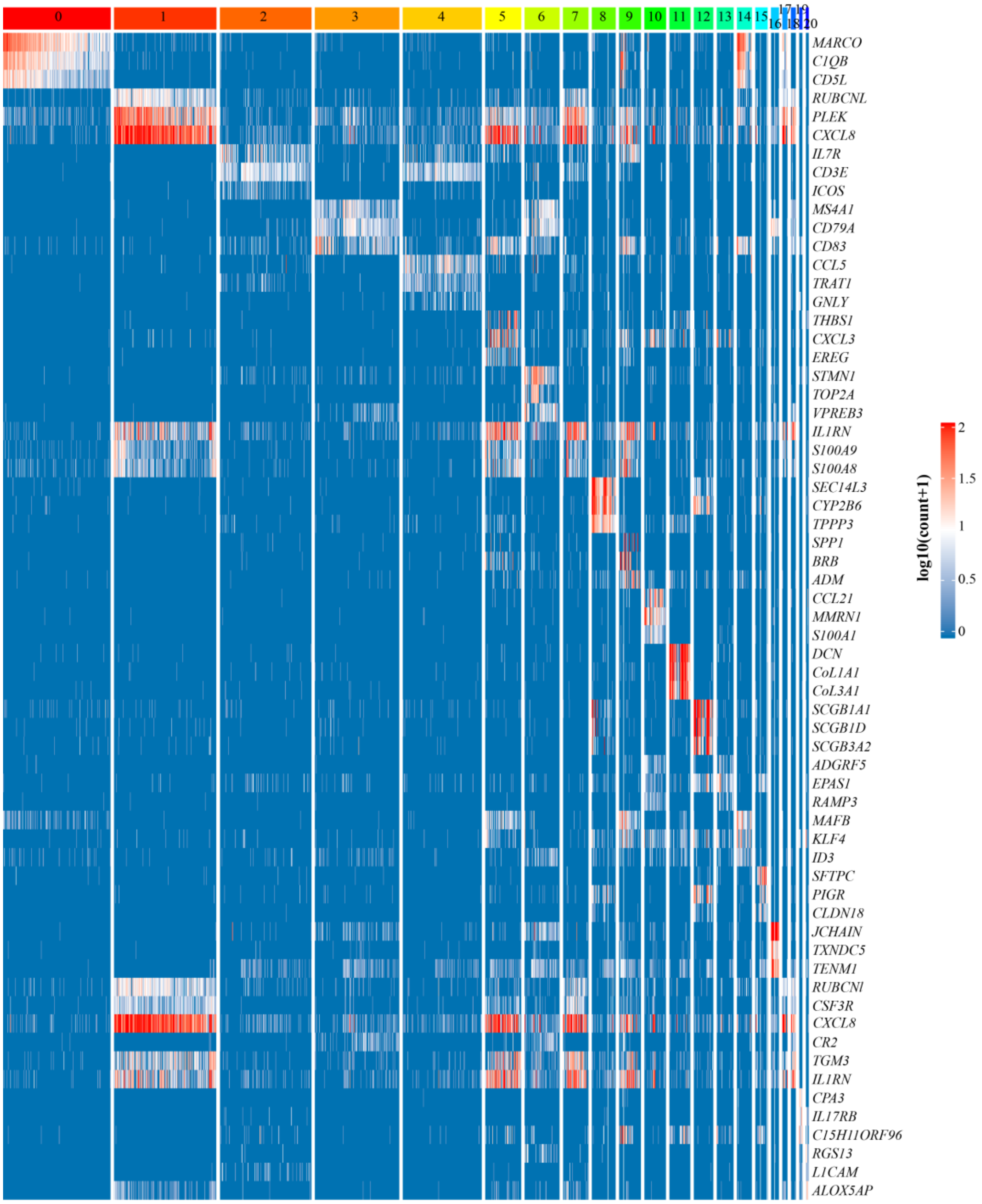

Supplementary Fig. 14. Expression of markers for each cluster in lung single cells of yak.

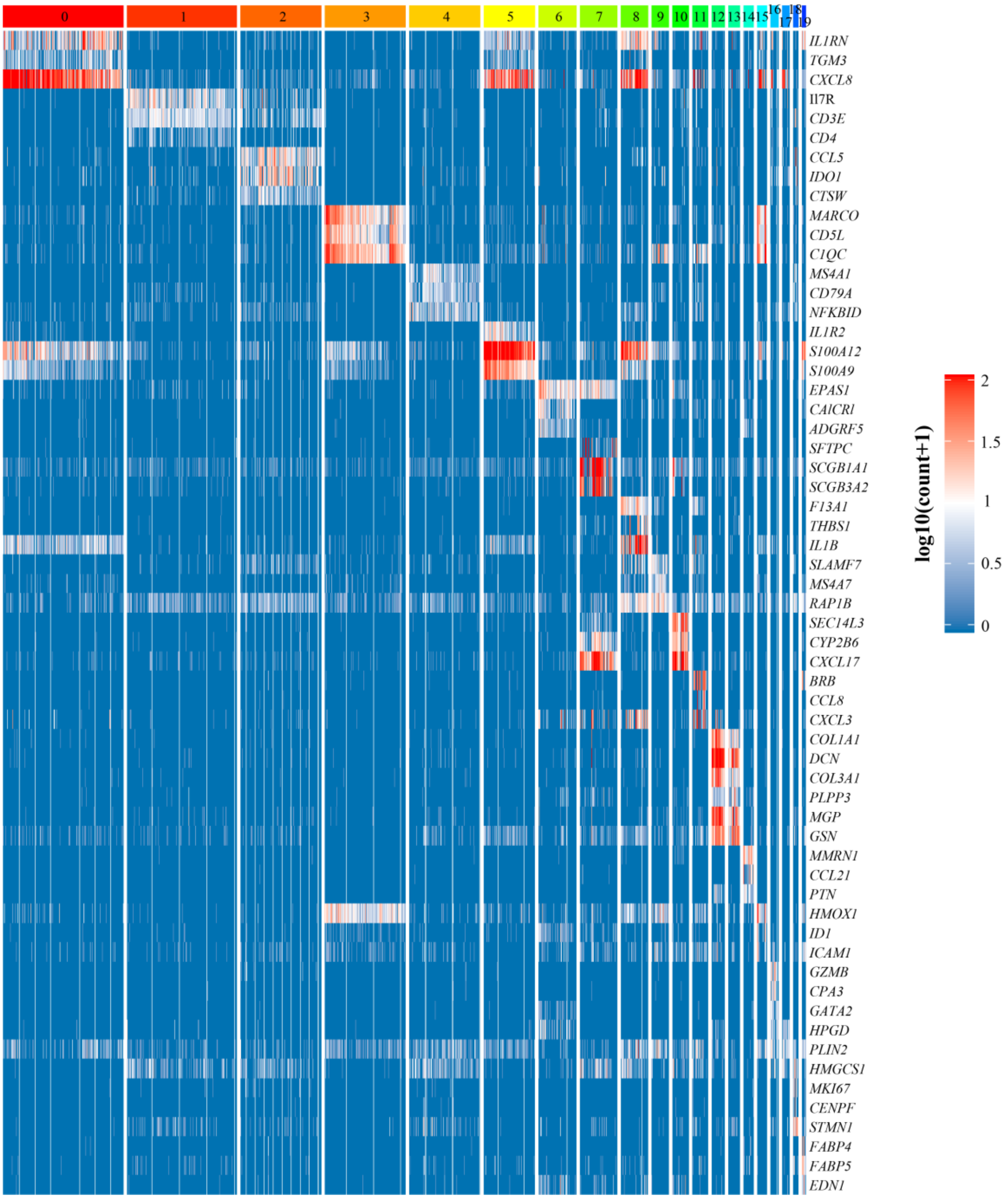

Supplementary Fig. 15. Expression of markers for each cluster in lung single cells of taurine cattle.

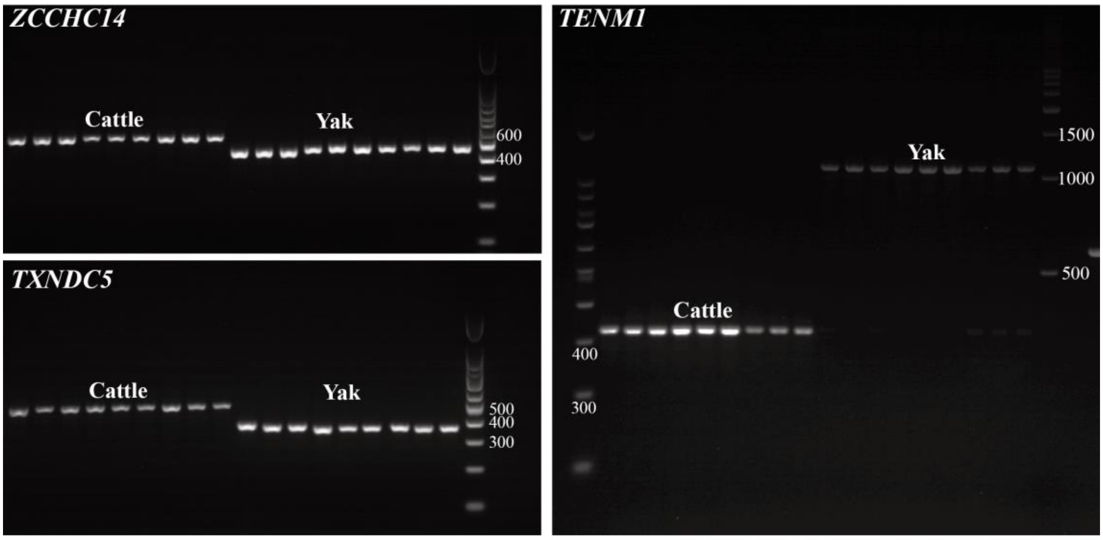

Supplementary Fig. 16. Validation of SVs in *TXNDC5*, *TENM1* and *ZCCHC14* gene by PCR. Note: Three biologically independent samples and three technical replicates were used for each gene in cattle (n=3 biologically independent samples) and yak (n=3 biologically independent samples) respectively. Source data are provided as a Source Data file.

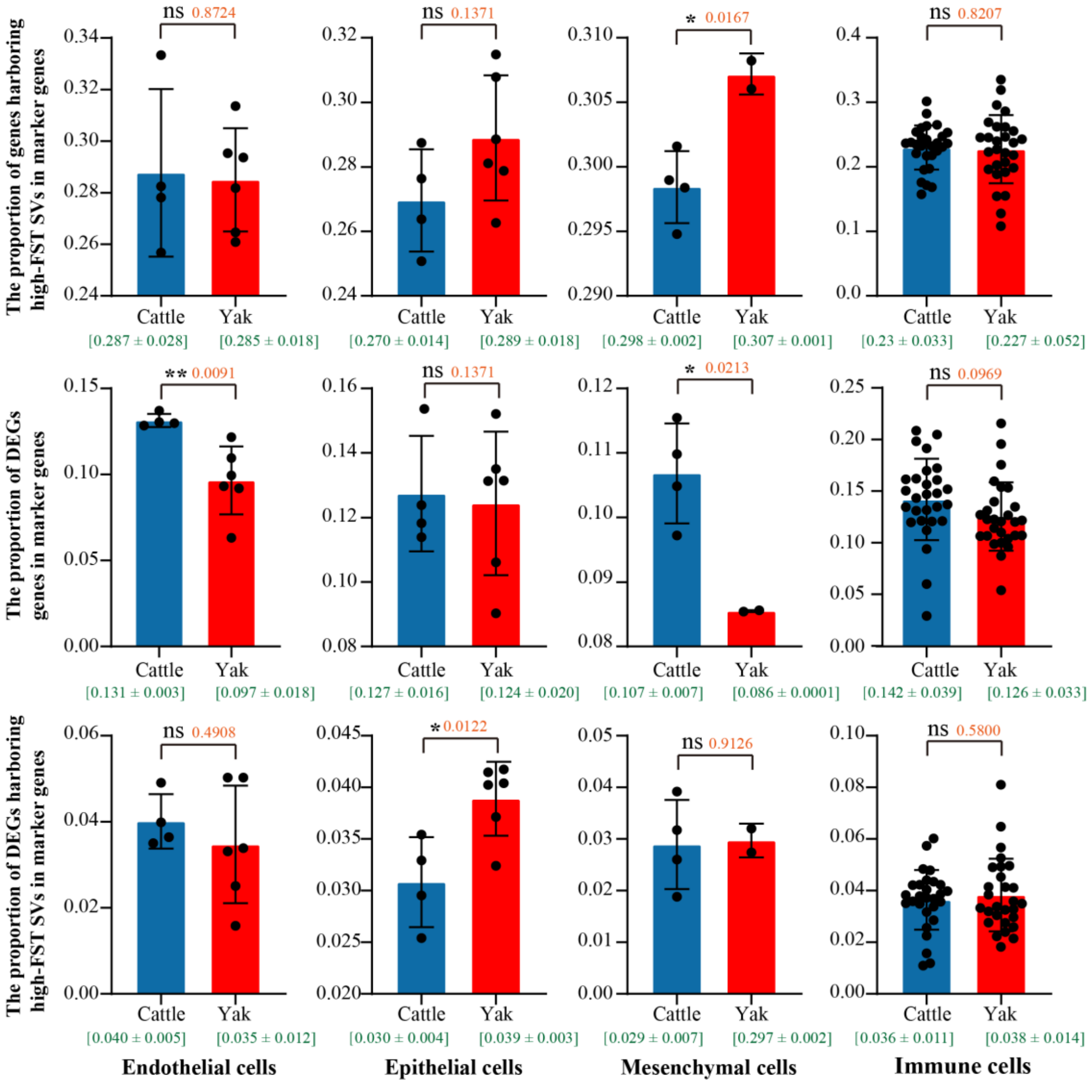

Supplementary Fig. 17. The Proportion of genes harboring high-FST SVs, DEGs and DEGs harboring high-FST SVs in marker genes of four cell types between domestic yak and taurine cattle. Note: Domestic yak (n=5 biologically independent samples) and taurine cattle (n=5 biologically independent samples) were used for analysis. Mean ± SD is shown in green and p-values are shown in orange (ns, no significance; *, *$p < 0.05$*; **, *$P<0.01$*). Source data are provided as a Source Data file.

Supplementary Table 1. Statistics of the Nanopore long reading sequencing data.

	Domestic yak	Wild yak
Active channels	2,817	2,758
Mean read length	17,769.9	11,533.6
Mean read quality	8.3	7.4
Median read length	17,318.0	9,430.0
Median read quality	8.9	8.0
Number of reads	8,844,845	16,575,499
Read length N50	23,712	20,197
Total bases	157,172,321,948	191,174,514,393

Supplementary Table 2. Summary of domestic yak genome sequencing data.

Pair-end libraries	Insert size	Total data (G)	Read length	Sequence coverage
Nanopore	-	157.17	-	52.39 X
Illumina reads	350bp	171.32	150bp	57.11 X
Hic	-	430.69	-	143.56 X
Total	-	759.18	-	253.06 X

Supplementary Table 3. Summary of wild yak genome sequencing data.

Pair-end libraries	Insert size	Total data (G)	Read length	Sequence coverage
Nanopore	-	191.17	-	63.72 X
Illumina reads	350bp	343.13	150bp	114.38 X
Hic	-	327.79	-	109.26 X
Total	-	862.09	-	287.36 X

Supplementary Table 4. Contig length and number of domestic yak.

Sample ID	Length		Number	
	Contig(bp)	Scaffold(bp)	Contig	Scaffold
Total	2,615,030,657	2,615,044,258	1,451	1,314
Max	134,830,518	156,616,011	-	-
Number >= 2000	-	-	1,447	1,310
N50	44,905,690	104,024,683	20	11
N60	36,922,725	81,896,857	26	14
N70	24,546,490	72,682,298	35	18
N80	16,532,916	63,654,981	48	21
N90	5,693,167	51,311,203	74	26

Supplementary Table 5. Contig length and number of wild yak.

Sample ID	Length		Number	
	Contig(bp)	Scaffold(bp)	Contig	Scaffold
Total	2,631,732,614	2,631,752,115	2,283	2,275
Max	136,014,743	155,998,435	-	-
Number >= 2000	-	-	2,252	2,244
N50	38,284,421	103,902,138	21	11
N60	28,251,563	81,554,258	29	14
N70	17,009,887	72,420,178	41	18
N80	10,978,447	68,837,362	60	21
N90	4,784,926	49,723,822	95	26

Supplementary Table 6. Genomic base content.

	Domestic yak		Wild yak	
	**Number (bp)**	**% of genome**	**Number (bp)**	**% of genome**
A	760,943,915	29.1	764,322,067	29.05
T	761,038,977	29.1	764,269,206	29.04
C	546,518,292	20.9	551,609,415	20.96
G	546,529,473	20.9	551,531,969	20.96
N	13,601	0	19,501	0
total	2,615,044,258	-	2,631,752,158	-
GC*	1,093,047,765	41.8	1,103,141,384	41.92

Supplementary Table 7. Statistics of genomic reads coverage.

		Domestic yak	Wild yak
**Reads**	**Mapping rate (%)**	**99.18%**	**99.35%**
Genome	Average sequencing depth	50.09X	62.46X
	Coverage	99.59%	99.19%
	Coverage_at_least_4X	99.47%	98.78%
	Coverage_at_least_10X	99.33%	97.89%
	Coverage_at_least_20X	98.07%	94.69%

Mapping rate: The proportion of reads compared to the genome; Average **sequencing depth**: The average depth at which each base in the genome is covered by reads; **Coverage:** The proportion of genomes covered by reads; **Coverage at least NX (%)**: The percentage of genomes covered by NX reads.

Supplementary Table 8. Number and percentage of genomic SNP.

	Domestic yak		Wild yak	
	**Number**	**Percentage (%)**	**Number**	**Percentage (%)**
All SNP	4,126,539	0.1594	3,559,760	0.138
Heterozygosis SNP	4,116,450	0.159	3,537,457	0.1371
Homology SNP	10,089	0.0004	22,303	0.0009

Supplementary Table 9. The results of genomic CEGMA evaluation of domestic yak and wild yak.

Species	Complete		Complete + Partial	
	#Prots	% completeness	#Prots	% completeness
Domestic yak	229	92.34	235	94.76
Wild yak	232	93.55	235	94.76

Note: Complete: core gene > 70% assembly; Complete+partial: The situation of partial assembly of core gene; #Prots: The number of core gene assembled; %completeness: The proportion of assembled core gene to core gene library.

Supplementary Table 10. The results of genomic BUSCO evaluation of domestic yak and wild yak.

Species	BUSCO notation assessment results
Domestic yak	C:93.3%[S:92.3%,D:1.0%],F:3.0%,M:3.7%,n:4104
Wild yak	C:93.5%[S:92.4%,D:1.1%],F:3.3%,M:3.2%,n:4104

Note: C-Complete BUSCOs; S-Complete and single-copy BUSCOs; D-Complete Duplicated BUSCOs; F-Fragmented BUSCOs; M-Missing BUSCOs; n-Total BUSCO searched.

Supplementary Table 11. Statistical results of genomic repeat sequences of domestic yak.

Type	Repeat Size(bp)	% of genome
Trf	36,910,668	1.41
Repeatmasker	1,170,782,734	44.77
Proteinmask	539,145,446	20.62
Total	1,194,277,221	45.67

Note: Total was the result of the above methods, and the non-redundant result after removing the overlap between them.

Supplementary Table 12. Statistics on the classification of genome repeat sequences in domestic Yak.

	Denovo+Repbase		TE Proteins		Combined TEs	
	Length (bp)	% in genome	Length (bp)	% in geno me	Length (bp)	% in genome
DNA	892,762	0.03	2,553,726	0.10	3,280,653	0.13
LINE	227,059,578	8.68	531,343,168	20.32	551,258,480	21.08
SINE	628,430	0.02	0	0	628,430	0.02
LTR	969,363,708	37.07	5,294,665	0.20	970,616,058	37.12
Unknown	22,641,265	0.87	0	0	22,641,265	0.87
Total	1,170,782,734	44.77	539,145,446	20.62	1,182,538,906	45.22

Note: Denovo+Repbase is a transposon element obtained by using the library predicted by RepeatModeler,RepeatScout,Piler and LTR_FINDER software combined with RepBase nucleic acid library, which was integrated by Uclust software according to 80Mel 80-80 principle and annotated by RepeatMasker software. TEproteins is the transposon element obtained by genome annotation by RepeatProteinMask software based on RepBase protein library, and CombinedTEs is the result of integration of the above two methods and de-redundancy. Unknown indicates that the repetitive sequence cannot be classified by RepeatMasker.

Supplementary Table 13. Statistics on the classification of genome repeat sequences in wild Yak.

Type	Repeat Size(bp)	% of genome
Trf	47,794,201	1.82
Repeatmasker	1,182,257,958	44.92
RepeatProteinMask	539,924,961	20.52
Total	1,205,480,278	45.81

Supplementary Table 14. Statistics on the Classification of Genome repeat sequences in wild Yak.

	Denovo+Repbase		TE Proteins		Combined TEs	
	Length (bp)	% in Genome	Length (bp)	% in Genome	Length (bp)	% in Genome
DNA	420,448	0.02	2,664,982	0.10	2,919,434	0.11
LINE	85,059,243	3.23	531,947,336	20.21	542,922,982	20.63
SINE	107,876	0.00	0	0	107,876	0.00
LTR	993,827,689	37.76	5,359,965	0.20	995,133,337	37.81
Unknown	145,931,877	5.55	0	0	145,931,877	5.55
Total	1,182,257,958	44.92	539,924,961	20.52	1,193,761,622	45.36

Supplementary Table 15. Statistical results of gene structure prediction of domestic yak

	Gene set	Number	Average transcript length (bp)	Average CDS length (bp)	Average exons per gene	Average exon length (bp)	Average intron length (bp)
De novo	Augustus	27,005	20,206.85	1,132.23	5.47	206.85	4,263.73
	GlimmerHMM	709,596	3,195.59	407.26	2.32	175.42	2,109.91
	SNAP	127,050	35,560.44	741.29	4.68	158.26	9,451.66
	Geneid	33,662	33,718.44	1,110.86	5.96	186.24	6,567.99
	Genscan	45,412	40,169.26	1,227.90	7.43	165.27	6,056.66
Homolog	Chi	19,733	27,047.22	1,469.68	8.12	181.05	3,593.60
	Oar	20,190	24,839.21	1,381.03	7.76	178.04	3,471.78
	Bbu	20,274	26,217.12	1,433.42	7.98	179.72	3,552.80
	Bta	19,790	24,731.15	1,413.32	7.87	179.49	3,392.14
	Rta	18,001	24,810.06	1,366.56	7.55	181.04	3,580.03
	Bbo	19,715	25,618.45	1,437.98	8.10	177.54	3,405.83
RNASeq	PASA	173,207	27,121.20	1,267.12	7.61	166.47	3,910.31
	Transcripts	35,637	60,423.41	4,503.61	11.34	397.27	5,409.93
EVM		34,612	20,225.61	1,067.93	5.72	186.74	4,059.96
Pasa-update*		34,153	24,970.53	1,125.99	6.02	186.89	4,745.40
Final set*		23,143	33,998.82	1,401.46	7.94	176.55	4,698.41

Note: * contains UTR areas, others are not. Bbu(*Bubalus_bubalis*); Bta(*Bos_taurus*); Oar(*Ovis_aries*); Chi(*Capra_hircus*); Bbo(*Bison_bonasus*); Rta(*Rangifer_tarandus*).

Supplementary Table 16. Statistical results of gene structure prediction of wild yak.

	**Gene set**	**Number**	**Average transcript length (bp)**	**Average CDS length (bp)**	**Average exons per gene**	**Average exon length (bp)**	**Average intron length (bp)**
De novo	Augustus	30,166	17,692.38	1,075.55	5.05	213.06	4,104.78
	GlimmerHMM	678,421	3,368.81	417.55	2.37	176.07	2,151.81
	SNAP	125,671	36,173.54	759.64	4.81	158.09	9,306.87
	Geneid	35,163	32,503.32	1,136.33	5.91	192.27	6,388.22
	Genscan	46,270	39,644.30	1,274.67	7.50	169.96	5,903.31
Homolog	Chi	19,787	26,807.74	1,473.33	8.11	181.63	3,562.41
	Oar	20,193	24,622.59	1,383.84	7.76	178.39	3,439.01
	Bbu	20,260	26,187.38	1,441.71	8.00	180.12	3,533.00
	Bta	19,816	24,573.64	1,417.65	7.88	179.80	3,363.55
	Rta	18,010	24,567.57	1,363.28	7.54	180.80	3,547.92
	Bbo	19,811	25,341.08	1,440.14	8.08	178.16	3,374.18
RNASeq	PASA	170,094	27,658.80	1,291.02	7.77	166.20	3,895.92
	Transcripts	35,616	60,446.70	4,530.41	11.44	396.15	5,358.01
EVM		37,918	18,385.19	1,040.54	5.37	193.81	3,970.05
Pasa-update*		37,408	22,842.86	1,097.15	5.67	193.67	4,661.33
Final set*		22,931	34,239.52	1,417.87	8.01	176.94	4,679.97

Note: Bbu(*Bubalus_bubalis*); Bta(*Bos_taurus*); Oar(*Ovis_aries*); Chi(*Capra_hircus*); Bbo(*Bison_bonasus*); Rta(*Rangifer_tarandus*). * contains UTR areas.

Supplementary Table 17. Statistical results of gene structure of closely related species.

Species	Number	Average transcript length (bp)	Average CDS length (bp)	Average exons per gene	Average exon length (bp)	Average intron length (bp)
Domestic yak	23,143	33,998.82	1,401.46	7.94	176.55	4,698.41
Rta	21,555	29,663.02	1,440.48	8.19	175.98	3,927.62
Bta	19,994	35,359.53	1,609.58	9.64	167.03	3,907.72
Bbo	21,542	31,457.97	1,572.14	9.35	168.16	3,579.63
Bbu	21,532	39,117.99	1,583.39	9.14	173.22	4,610.64
Chi	20,587	44,038.39	1,689.91	9.55	176.95	4,952.89
Oar	20,921	35,305.75	1,559.03	9.62	162.13	3,916.74

Note: Bbu(*Bubalus_bubalis*); Bta(*Bos_taurus*); Oar(*Ovis_aries*); Chi(*Capra_hircus*); Bbo(*Bison_bonasus*); Rta(*Rangifer_tarandus*).

Supplementary Table 18. Gene structure analysis of closely related species.

Species	Number	Average transcript length (bp)	Average CDS length (bp)	Average exons per gene	Average exon length (bp)	Average intron length (bp)
Wild yak	22,931	34,239.52	1,417.87	8.01	176.94	4,679.97
Rta	21,555	29,663.02	1,440.48	8.19	175.98	3,927.62
Bta	19,994	35,359.53	1,609.58	9.64	167.03	3,907.72
Bbo	21,542	31,457.97	1,572.14	9.35	168.16	3,579.63
Bbu	21,532	39,117.99	1,583.39	9.14	173.22	4,610.64
Chi	20,587	44,038.39	1,689.91	9.55	176.95	4,952.89
Oar	20,921	35,305.75	1,559.03	9.62	162.13	3,916.74

Note: Bbu(*Bubalus_bubalis*); Bta(*Bos_taurus*); Oar(*Ovis_aries*); Chi(*Capra_hircus*); Bbo(*Bison_bonasus*); Rta(*Rangifer_tarandus*).

Supplementary Table 19. Gene function annotation.

	Domestic yak		Wild yak	
	**Number**	**Percentage (%)**	**Number**	**Percentage (%)**
Total	23,143	-	22,931	-
Swissprot	20,877	90.2	20,754	90.5
Nr	22,001	95.1	21,834	95.2
KEGG	19,121	82.6	19,040	83
InterPro	20,541	88.8	20,443	89.2
GO	14,660	63.3	14,694	64.1
Pfam	17,985	77.7	18,024	78.6
Annotated	22,056	95.3	21,884	95.4
Unannotated	1,087	4.7	1,047	4.6

Supplementary Table 20. Statistical results of genome non-coding RNA of domestic yak.

Type		Copy number	Average length (bp)	Total length (bp)	% of genome
miRNA		9,642	95.10	916,980	0.035066
tRNA		34,835	72.83	2,537,004	0.097016
rRNA	rRNA	960	217.21	208,522	0.007974
	18S	97	515.85	50,037	0.001913
	28S	311	344.48	107,132	0.004097
	5.8S	31	134.58	4,172	0.000160
	5S	521	90.56	47,181	0.001804
snRNA	snRNA	1,981	115.78	229,359	0.008771
	CD-box	316	94.10	29,737	0.001137
	HACA-box	385	134.49	51,779	0.001980
	splicing	1,222	113.74	138,988	0.005315
	scaRNA	49	169.27	8,294	0.000317
	Unknown	9	62.33	561	0.000021

Supplementary Table 21. Statistical results of genome non-coding RNA of wild yak.

Type		Copy number	Average length (bp)	Total length (bp)	% of genome
miRNA		9,635	95.14	916,693	0.034832
tRNA		34,900	72.82	2,541,416	0.096567
rRNA	rRNA	770	135.19	104,099	0.003956
	18S	74	263.30	19,484	0.000740
	28S	187	214.35	40,084	0.001523
	5.8S	11	111.09	1,222	0.000046
	5S	498	86.97	43,309	0.001646
snRNA	snRNA	1,984	115.84	229,821	0.008733
	CD-box	321	95.68	30,712	0.001167
	HACA-box	386	135.03	52,122	0.001981
	splicing	1,221	113.36	138,416	0.005259
	scaRNA	47	170.43	8,010	0.000304
	Unknown	9	62.33	561	0.000021

Supplementary Table 22. VEP annotation results of 11 SVs causing frameshift variants.

Location	SVtype	SYMBOL	Gene
1:70466529-70466578	deletion	*MUC20*	ENSBTAG00000032819
10:101591887-101591928	deletion	*EFCAB11*	ENSBTAG00000013136
12:36413656-36413778	deletion	*PARP4*	ENSBTAG00000025400
12:86636566-86636667	deletion	-	ENSBTAG00000048756
17:72979092-72979127	deletion	*DGCR8*	ENSBTAG00000019869
2:77262893-77263026	deletion	-	ENSBTAG00000051403
2:135317368-135317427	deletion	*PADI3*	ENSBTAG00000012043
21:41268601-41268642	deletion	*SCFD1*	ENSBTAG00000017565
26:23340078-23340244	deletion	*SFXN2*	ENSBTAG00000004321
3:17212873-17212982	deletion	*LORICRIN*	ENSBTAG00000048718
4:119095352-119095414	deletion	*PTPRN2*	ENSBTAG00000004958
7:17104672-17104859	deletion	*PRAM1*	ENSBTAG00000021041
8:11015253-11015311	deletion	*ESCO2*	ENSBTAG00000006551

Supplementary Table 23. Information of public data used for transcriptome analysis.

	Species	Organization
SRR3109702	cattle	heart
SRR3109703	cattle	heart
SRR3109704	cattle	heart
SRR3109708	cattle	kidney
SRR3109709	cattle	kidney
SRR3109710	cattle	kidney
SRR3109705	cattle	liver
SRR3109706	cattle	liver
SRR3109707	cattle	liver
SRR5190375	cattle	muscle
SRR5190377	cattle	muscle
SRR5190379	cattle	muscle
SRR5190450	cattle	spleen
SRR5190451	cattle	spleen
SRR5190452	cattle	spleen
SRR5190423	yak	heart
SRR5190428	yak	heart
SRR5190431	yak	heart
SRR5190507	yak	kidney
SRR5190508	yak	kidney
SRR5190509	yak	kidney
SRR5190489	yak	liver
SRR5190491	yak	liver
SRR5190493	yak	liver
SRR5190424	yak	muscle
SRR5190426	yak	muscle
SRR5190429	yak	muscle
SRR5190490	yak	spleen
SRR5190492	yak	spleen
SRR5190494	yak	spleen
DY_Heart	yak	heart
DY_Kidney	yak	kidney
DY_Liver	yak	liver
DY_Lung	yak	lung
DY_Muscle	yak	muscle
DY_spleen	yak	spleen
SRR10174899	cattle	lung
SRR10174900	cattle	lung
SRR10174901	cattle	lung

SRR10174903	cattle	lung
	**Species**	**Organization**
SRR12165296	yak	lung
SRR12165297	yak	lung
SRR12165298	yak	lung
SRR12165299	yak	lung
SRR12165300	yak	lung
SRR12697118	cattle	lung
SRR12697119	cattle	lung

Supplementary Table 24. Identification and expression of yak-specific genes in yak lung

geneID	gene_name	Normalized_count
evm.model.HIC_ASM_14.883	*POTEA*	99.36979
evm.model.HIC_ASM_28.450	*TGas006m08.1*	92.22537
evm.model.HIC_ASM_14.1435	*UGT1A10*	69.08929
evm.model.HIC_ASM_26.1082	*SIGLECL1*	55.5139
evm.model.HIC_ASM_13.589	*ADAT3*	37.9541
evm.model.HIC_ASM_13.1513	*Hist1h2ap*	31.82022
evm.model.HIC_ASM_28.63	*HSFX4*	31.51667
evm.model.HIC_ASM_9.1510	*EFCAB13*	20.8685
evm.model.HIC_ASM_28.412	*SMIM10L2B*	19.17757
evm.model.HIC_ASM_14.1433	*UGT1A3*	16.20383
evm.model.HIC_ASM_25.254	*GCKR*	15.86087
evm.model.HIC_ASM_14.895	*COL24A1*	11.23844
evm.model.HIC_ASM_15.641	*COL22A1*	10.76062
evm.model.CTG579.1	*ANKRD19P*	9.201478
evm.model.HIC_ASM_19.124	*ZNF607*	7.832466
evm.model.CTG442.1	*TRDV1*	6.611976
evm.model.HIC_ASM_5.34	*COL19A1*	3.976988
evm.model.CTG310.1	*CCDC144A*	3.841086
evm.model.HIC_ASM_25.256	*C2orf16*	3.582724
evm.model.HIC_ASM_24.649	*FAM240A*	2.900227
evm.model.HIC_ASM_28.1072	*RTL4*	1.752299
evm.model.HIC_ASM_23.869	*COL25A1*	1.22
evm.model.HIC_ASM_5.604	*ULBP3*	0.917116
evm.model.HIC_ASM_8.118	*Sgs4*	0.759679
evm.model.HIC_ASM_28.895	*PELPK1*	0.668683
evm.model.CTG1165.1	*TRAV26-1*	0.593419
evm.model.CTG245.4	*TRAV14DV4*	0.491737
evm.model.HIC_ASM_23.426	*NOS*	0.468436

Supplementary Table 25. 127 DEGs carrying high-FST SVs within the exonic and promoter region.

gene name			
ENSBTAG00000054546	*ZNF691*	*DUSP28*	*PPP1R16B*
ENSBTAG00000053674	*ZNF341*	*DENND3*	*PLA2G4A*
ENSBTAG00000053499	*ZNF143*	*DDRGK1*	*PKD1L3*
ENSBTAG00000053457	*ZBP1*	*CTTNBP2NL*	*PIGA*
ENSBTAG00000053272	*XPC*	*CSTF3*	*PI4KA*
ENSBTAG00000052982	*WDR5*	*CSRP3*	*PCCA*
ENSBTAG00000052608	*VAV3*	*CPS1*	*PADI3*
ENSBTAG00000052342	*UBTD1*	*CDH5*	*NUP210L*
ENSBTAG00000052053	*U6*	*CDC25C*	*NUP155*
ENSBTAG00000051677	*TMEM229A*	*CD82*	*NFIC*
ENSBTAG00000050645	*SV2A*	*CD320*	*NAXD*
ENSBTAG00000050606	*SPARCL1*	*CCL16*	*NAGK*
ENSBTAG00000049808	*SMYD4*	*CCL14*	*MEF2C*
ENSBTAG00000048417	*SLC9A5*	*CATSPERD*	*MED31*
ENSBTAG00000047073	*SLC41A3*	*CAT*	*MAST3*
ENSBTAG00000045531	*SHISA4*	*BRF1*	*LGMN*
ENSBTAG00000042605	*SH2B2*	*BLCAP*	*KLHL40*
ENSBTAG00000042478	*SFXN2*	*BANK1*	*KLHDC7A*
ENSBTAG00000039671	*SFTPD*	*ATG16L1*	*KIF13A*
ENSBTAG00000039499	*SEMA4C*	*ASCC2*	*IL2RB*
ENSBTAG00000038064	*RXRA*	*ARSI*	*IKZF2*
ENSBTAG00000037799	*RORA*	*ARPC1A*	*IGF2R*
ENSBTAG00000027962	*RNASE10*	*AKT1*	*IFI47*
ENSBTAG00000022275	*RHEB*	*AGPAT5*	*IFFO2*
ENSBTAG00000014862	*RGS9*	*AGPAT2*	*HHATL*
ENSBTAG00000006398	*RGS19*	*ADPRS*	*GULO*
ENSBTAG00000004920	*RASA3*	*ADAMTS14*	*GSDMD*
ENSBTAG00000004109	*RAF1*	*ADAM22*	*GPR137B*
GP5	*RAB1A*	*ACO1*	*GPR132*
GLTP	*PTPRK*	*ABR*	*GPKOW*
FPGS	*FOXO6*	*FMO5*	*FICD*
FBXO30	*F8*	*ENTPD5*	

Supplementary Table 26. Primers used in this study.

Gene	Sequence (5'to3')
ZCCHC14	AGGAACAGCACTAAGCGTCC
	CCCGTCTCTGTTGAGAACCC
TXNDC5	CTGCCCCTCTCTGATAACGC
	GCTGGATAGTCACGCCTTGT
TENM1	TCACTACAGACAGGTGGCTTC
	AGAAAGCTTCAGCAGTTCATTTTG
