# Supplementary material for: Long read genome assemblies complemented by single cell RNA-sequencing reveal genetic and cellular mechanisms underlying the adaptive evolution of yak
Source: Nat Commun. 2022 Sep 6;13:4887. doi: 10.1038/s41467-022-32164-9 (PMC9448747; doi:10.1038/s41467-022-32164-9)
Supplement: Supplementary file 2 — Description of Additional Supplementary Files [file 41467_2022_32164_MOESM2_ESM.pdf]

Title: Supplementary Data 1:
Description: The annotation results of High-Fst SVs by VEP. SVs were detected by long-read resequencing data of cattle and yak (3 taurine cattle, 19 domestic yak and 7 wild yak), related to Fig. 1.

Title: Supplementary Data 2:
Description: The one-way ANOVA and Tukey's multiple comparisons results of SV-carrying Differentially expressed genes (DEGs) of six tissues. DEGs identified from bulk RNA-Seq analysis of yak and taurine cattle (3 taurine cattle and 4 yak for heart, spleen, kidney, muscle, and liver. 5 taurine cattle and 5 yak for lung), related to Fig. 2a.

Title: Supplementary Data 3:
Description: The chi-square test results of differentially expressed genes (DEGs) in six tissues and SV-carrying genes, related to Fig. 2a. DEGs identified from bulk RNA-Seq analysis of yak and taurine cattle (3 taurine cattle and 4 yak for heart, spleen, kidney, muscle, and liver. 5 taurine cattle and 5 yak for lung) related to Fig. 2a.

Title: Supplementary Data 4:
Description: The motif enrichment results of the peak located in promoter carrying high-FST SVs by MEME. Peaks were detected by ATACseq data of lung tissue from two cattle, related to Fig. 2c.

Title: Supplementary Data 5:
Description: The correlation values for each of yak and taurine cattle cell subgroups (5 taurine cattle and 5 yak). Calculate the Pearson correlation coefficient by the corr.test function, related to Fig. 3c.

Title: Supplementary Data 6:
Description: Differentially expressed genes in mesenchymal cells from scRNA-Seq analysis of yak and taurine cattle (5 taurine cattle and 5 yak). Cut-off for DEGs is adjusted P-value < 0.05 (MAST differential expression test with Bonferroni correction), related to Fig. 4a.

Title: Supplementary Data 7:
Description: The GO enrichment results of Differentially expressed genes (DEGs). DEGs identified from scRNA-Seq analysis of yak vs taurine cattle (5 taurine cattle and 5 yak) in Mesenchymal Cells. Cut-off for DEGs is adjusted P-value < 0.05 (the g:SCS correction method), related to Fig. 4a.
