# Supplementary material for: Long read genome assemblies complemented by single cell RNA-sequencing reveal genetic and cellular mechanisms underlying the adaptive evolution of yak
Source: Nat Commun. 2022 Sep 6;13:4887. doi: 10.1038/s41467-022-32164-9 (PMC9448747; doi:10.1038/s41467-022-32164-9)
Supplement: Supplementary file 10 — Reporting Summary [file 41467_2022_32164_MOESM10_ESM.pdf]

nature portfolio

Corresponding author(s): Prof. Xin-Quan Zhao, Dong-Dong Wu, and Qi-En Yang

Last updated by author(s): Jun 28, 2022

Reporting Summary

Nature Portfolio wishes to improve the reproducibility of the work that we publish. This form provides structure for consistency and transparency in reporting. For further information on Nature Portfolio policies, see our Editorial Policies and the Editorial Policy Checklist.

Statistics

For all statistical analyses, confirm that the following items are present in the figure legend, table legend, main text, or Methods section.

n/a	Confirmed	
☐	☒	The exact sample size (*n*) for each experimental group/condition, given as a discrete number and unit of measurement
☐	☒	A statement on whether measurements were taken from distinct samples or whether the same sample was measured repeatedly
☐	☒	The statistical test(s) used AND whether they are one- or two-sided<br>*Only common tests should be described solely by name; describe more complex techniques in the Methods section.*
☒	☐	A description of all covariates tested
☒	☐	A description of any assumptions or corrections, such as tests of normality and adjustment for multiple comparisons
☐	☒	A full description of the statistical parameters including central tendency (e.g. means) or other basic estimates (e.g. regression coefficient) AND variation (e.g. standard deviation) or associated estimates of uncertainty (e.g. confidence intervals)
☐	☒	For null hypothesis testing, the test statistic (e.g. *F*, *t*, *r*) with confidence intervals, effect sizes, degrees of freedom and *P* value noted<br>*Give P values as exact values whenever suitable.*
☒	☐	For Bayesian analysis, information on the choice of priors and Markov chain Monte Carlo settings
☒	☐	For hierarchical and complex designs, identification of the appropriate level for tests and full reporting of outcomes
☐	☒	Estimates of effect sizes (e.g. Cohen's *d*, Pearson's *r*), indicating how they were calculated

Our web collection on statistics for biologists contains articles on many of the points above.

Software and code

Policy information about availability of computer code

Data collection	Two raw genome sequencing datas were collected by using PromethION platform; Two raw Hic datas were produced by Illumina PE150, And Four raw single-cell RNA-sequencing datas originated from BD Rhapsody platform.
Data analysis	The following softwares were used in data analysis: wtdbg2 (v2.5), Racon (v1.3.1), Pilon (v1.22),Trinity (2.1.1), all-hic (v0.9.8), Merqury (v1.3), RepeatModeler (v2.0.1), RepeatMasker (v4.1.0), GeneWise (v2.4.1), Augustus (v3.3.2), Geneid (v1.4), Genescan (v1.0), GlimmerHMM (v3.0.4), Snap (v2013.11.29), Hisat (v2.0.4), Stringtie (v1.3.3), EVidenceModeler (v1.1.1), Blastp (v2.2.26), InterProScan (v5.35), tRNAscan-SE (v1.4), infernal (v1.1.2), NGMLR (v0.2.7), cuteSV (v1.0.11), SURVIVOR (v1.0.7), vcftools (v0.1.17), hisat2 (v2.2.1), DESeq2 (v1.30.1), ggplot2 (v3.3.3), Miropeats (v2.02), Graphpad prism(v8.0), Orthofinder (v2.5.4), STAR (v2.7.1a), Seurat (v3.2.0), ComplexHeatmap (v2.6.2), GeneOverlap (v1.22.0) and psych (v2.0.8)

For manuscripts utilizing custom algorithms or software that are central to the research but not yet described in published literature, software must be made available to editors and reviewers. We strongly encourage code deposition in a community repository (e.g. GitHub). See the Nature Portfolio guidelines for submitting code & software for further information.

Data

Policy information about availability of data

All manuscripts must include a data availability statement. This statement should provide the following information, where applicable:

- Accession codes, unique identifiers, or web links for publicly available datasets
- A description of any restrictions on data availability
- For clinical datasets or third party data, please ensure that the statement adheres to our policy

We have deposited the assembled genome, raw Hic data and raw Nanopore data of wild yak and domestic yak at Sequence Read Archive (SRA) database of National

Center for Biotechnology Information (NCBI) database with accession BioProject codes: PRJNA720245 and PRJNA720246. And the lung single-cell RNA-Sequencing data of domestic yak and taurine cattle have been at the NCBI database with accession BioProject codes: PRJNA720247 and PRJNA720248.

Field-specific reporting

Please select the one below that is the best fit for your research. If you are not sure, read the appropriate sections before making your selection.

- [x] Life sciences
- [ ] Behavioural & social sciences
- [ ] Ecological, evolutionary & environmental sciences

For a reference copy of the document with all sections, see nature.com/documents/nr-reporting-summary-flat.pdf

Life sciences study design

All studies must disclose on these points even when the disclosure is negative.

Sample size	For reference genome construction, one wild yak and one domestic yak were used. For single-cell RNA sequencing, we collected the lung tissue samples from five adult male taurine cattle and five adult male yak. For SV detection, data from 3 taurine cattle, 19 domestic yak and 7 wild yak were used. All statistically significant differences are reported with corresponding P-values.
Data exclusions	For single cell-RNA seq data analysis,we excluded low quality cells through quality control pipeline. In Fig. 4, we only used mesenchymal cells for further analysis, the other cell types were excluded.
Replication	Lung tissue structure of yak (n=5) and taurine cattle (n=3) were confirmed by H&E and elastic fiber staining. Representative images were provided in the figure 4c. Results in Fig.4d , and Fig.4e were performed in three experimental replicates.
Randomization	We randomly selected samples from hundreds of the yak and taurine cattle individuals.
Blinding	H&E and elastic fiber staining results were assessed by two independent authors who were blinded to group allocation during data analysis.

Reporting for specific materials, systems and methods

We require information from authors about some types of materials, experimental systems and methods used in many studies. Here, indicate whether each material, system or method listed is relevant to your study. If you are not sure if a list item applies to your research, read the appropriate section before selecting a response.

Materials & experimental systems

n/a	Involved in the study	
[x]	[ ]	Antibodies
[x]	[ ]	Eukaryotic cell lines
[x]	[ ]	Palaeontology and archaeology
[ ]	[x]	Animals and other organisms
[x]	[ ]	Human research participants
[x]	[ ]	Clinical data
[x]	[ ]	Dual use research of concern

Methods

n/a	Involved in the study	
[x]	[ ]	ChIP-seq
[x]	[ ]	Flow cytometry
[x]	[ ]	MRI-based neuroimaging

Animals and other organisms

Policy information about studies involving animals; ARRIVE guidelines recommended for reporting animal research

Laboratory animals	The samples of yak and taurine cattles lungs were collected from local slaughterhouse. We did not adjust our analysis for age and gen der. Details samples processing steps a re provided in the materials and methods.
Wild animals	An adult male wild yak raised at an altitude of 4200 meters in Qumalai County, Qinghai Province. Only minimal amounts of blood were sampled from each animal which were then immediately returned to the herd.
Field-collected samples	No field-collected samples were used.
Ethics oversight	Animal experiments were approved by the Animal Ethics and Welfare Committee at Northwest Institute of Plateau Biology, Chinese Academy of Sciences.

Note that full information on the approval of the study protocol must also be provided in the manuscript.
